# Oncolytic herpesvirus expressing PD-L1 BiTE for cancer therapy: exploiting tumor immune suppression as an opportunity for targeted immunotherapy

**DOI:** 10.1136/jitc-2020-001292

**Published:** 2021-04-05

**Authors:** Hena Khalique, Richard Baugh, Arthur Dyer, Eleanor M. Scott, Sally Frost, Sarah Larkin, Janet Lei-Rossmann, Leonard W. Seymour

**Affiliations:** Department of Oncology, University of Oxford, Oxford, Oxfordshire, UK

**Keywords:** B7-H1 antigen, immunotherapy, oncolytic virotherapy, tumor microenvironment, T-lymphocytes

## Abstract

**Background:**

Programmed death-ligand 1 (PD-L1) is an important immune checkpoint protein that can be regarded as a pan-cancer antigen expressed by multiple different cell types within the tumor. While antagonizing PD-L1 is well known to relieve PD-1/PD-L1-mediated T cell suppression, here we have combined this approach with an immunotherapy strategy to target T cell cytotoxicity directly toward PD-L1-expressing cells. We developed a bi-specific T cell engager (BiTE) crosslinking PD-L1 and CD3ε and demonstrated targeted cytotoxicity using a clinically relevant patient-derived ascites model. This approach represents an immunological ‘volte-face’ whereby a tumor immunological defense mechanism can be instantly transformed into an Achilles’ heel for targeted immunotherapy.

**Methods:**

The PD-L1 targeting BiTE comprises an anti-PD-L1 single-chain variable fragment (scFv) or nanobody (NB) domain and an anti-CD3 scFv domain in a tandem repeat. The ability to activate T cell cytotoxicity toward PD-L1-expressing cells was established using human carcinoma cells and PD-L1-expressing human (‘M2’) macrophages in the presence of autologous T cells. Furthermore, we armed oncolytic herpes simplex virus-1 (oHSV-1) with PD-L1 BiTE and demonstrated successful delivery and targeted cytotoxicity in unpurified cultures of malignant ascites derived from different cancer patients.

**Results:**

PD-L1 BiTE crosslinks PD-L1-positive cells and CD3ε on T cells in a ‘pseudo-synapse’ and triggers T cell activation and release of proinflammatory cytokines such as interferon-gamma (IFN-γ), interferon gamma-induced protein 10 (IP-10) and tumour necrosis factor-α (TNF-α). Activation of endogenous T cells within ascites samples led to significant lysis of tumor cells and M2-like macrophages (CD11b+CD64+ and CD206+/CD163+). The survival of CD3+ T cells (which can also express PD-L1) was unaffected. Intriguingly, ascites fluid that appeared particularly immunosuppressive led to higher expression of PD-L1 on tumor cells, resulting in improved BiTE-mediated T cell activation.

**Conclusions:**

The study reveals that PD-L1 BiTE is an effective immunotherapeutic approach to kill PD-L1-positive tumor cells and macrophages while leaving T cells unharmed. This approach activates endogenous T cells within malignant ascites, generates a proinflammatory response and eliminates cells promoting tumor progression. Using an oncolytic virus for local expression of PD-L1 BiTE also prevents ‘on-target off-tumor’ systemic toxicities and harnesses immunosuppressive protumor conditions to augment immunotherapy in immunologically ‘cold’ clinical cancers.

## Background

Oncolytic viruses (OVs) replicate selectively within tumor cells and lyse them. They can also be armed to provide tumor-selective expression of therapeutic biologicals, including antibodies recognizing tumor antigens to promote antibody-dependent cellular cytotoxicity or checkpoint inhibitor antibodies designed to modulate the local immune microenvironments.^1–6^ OVs have also been engineered to express bispecific T cell engagers (BiTEs),^7–9^ comprised of two single-chain variable fragments (scFvs) that recognize a target antigen along with T cell receptor (usually CD3ε) to mediate non-physiological cell interactions as a form of targeted immunotherapy.^10^ BiTEs induce formation of a pseudosynapse between T cells and cells expressing the target antigen, mediating CD3 clustering and T cell activation for antigen-specific cytotoxicity. As well as the FDA-approved CD19-targeting BiTE, blinatumomab,^11^ many other BiTEs are in preclinical trials and are demonstrating promising results.^9 12^

BiTEs provide a powerful strategy to mediate tumor-targeted cytotoxicity and are ideally suited for expression from OVs locally within the tumor microenvironment (TME), since intravenous delivery of free BiTEs may trigger ‘on-target, off-tumor’ cytotoxicity. The first generation of BiTEs targeted antigens associated with the surface of tumor cells, thereby recognizing the same cell population as the OV.^7 13 14^ Subsequent generations targeted antigens expressed on stromal cells such as tumor-associated fibroblasts, providing a strategy whereby the OV and activated T cells are cytotoxic to different cell populations within the tumor.^9 12^ Here we have taken this concept further and designed an OV encoding a BiTE targeting the pan-cancer antigen, programmed death-ligand 1 (PD-L1). PD-L1 is often expressed on tumor parenchymal cells, tumor-associated monocytes and macrophages (TAM) and neutrophils^15–18^ and can cause tumor immune suppression by inhibiting T cell activation via PD-1/PD-L1 signaling.^19 20^

We explore approaches to direct T cell cytotoxicity toward PD-L1 to mediate broad cytotoxicity in the TME. We develop a BiTE targeting PD-L1 and encode it within oncolytic herpes simplex virus-1 (oHSV-1).^3 21^ Using a human malignant ascites model, we demonstrate PD-L1 BiTE activates tumor-associated T cells resulting in depletion of tumor cells and macrophages. Strikingly, this approach capitalizes on ascites-mediated upregulation of PD-L1 and is potentiated, rather than inhibited, in this immunosuppressive microenvironment.

## Methods

### Cell lines and cell culture

HEK293A, DLD-1 and Vero cells (ATCC) were cultured and maintained in Dulbecco’s modified Eagle medium (DMEM, Sigma-Aldrich, UK) supplemented with 10% (v/v) heat-inactivated fetal bovine serum (FBS, Gibco, UK). Viral infections and propagations were performed in DMEM with 2% (v/v) FBS.

### Engineering and production of BiTE

**Figure 1:**
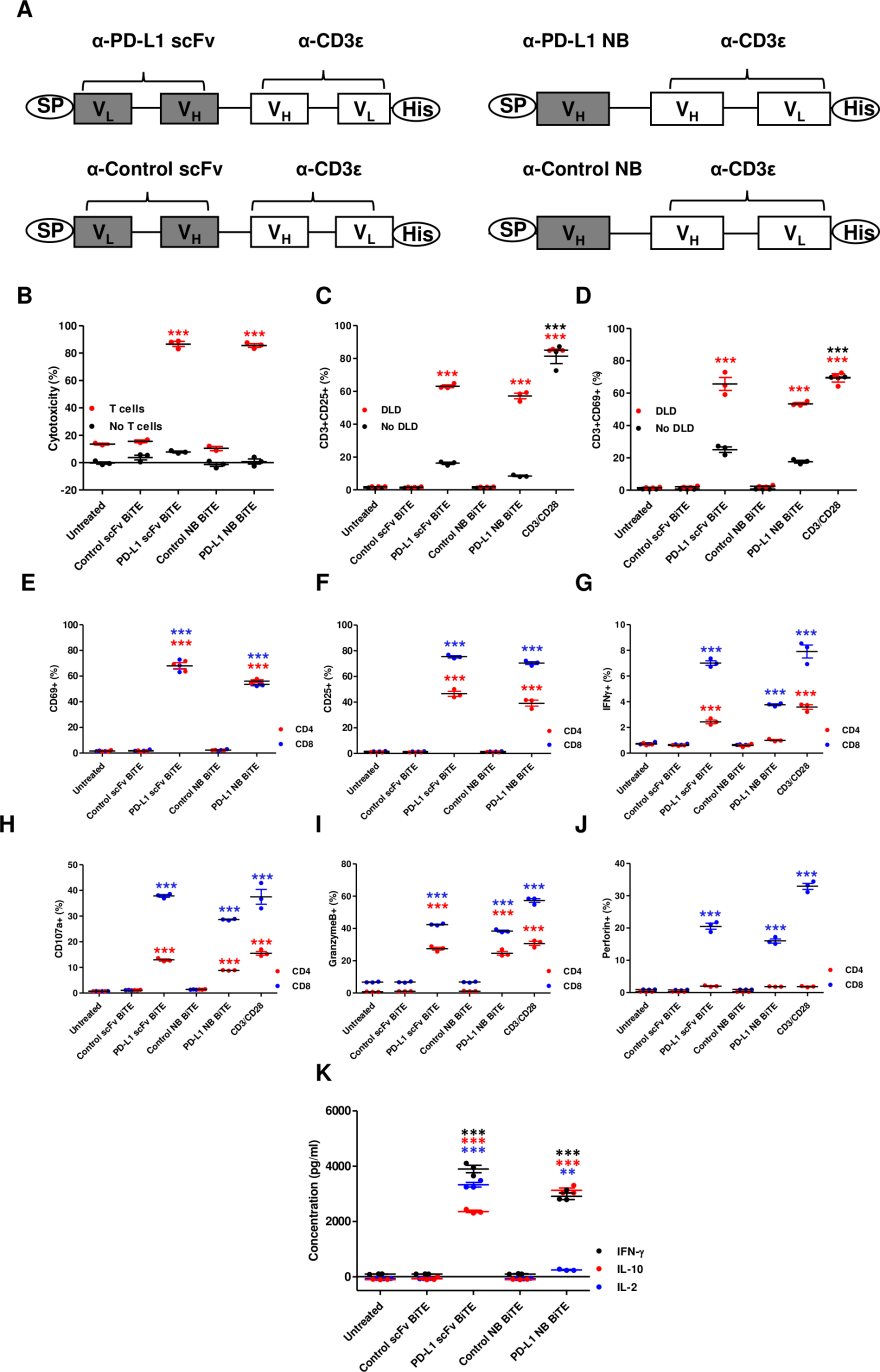
*In vitro* characterization of PD-L1 targeting BiTE. (A) Schematic representation of PD-L1 BiTEs and control BiTEs. VL and VH domains of single-chain variable fragment (scFv) or VH domain of nanobody (NB) targeting PD-L1 or irrelevant antigens were linked to VH and VL domains of anti-CD3ε scFv by flexible glycine-serine linkers. An immunoglobulin signal peptide (SP) and hexahistidine (His) affinity tag are added at N-terminal and C-terminal, respectively. PBMC-derived T cells were directed to kill DLD-1 carcinoma cells (5:1) using PD-L1 BiTEs at a dose of 40 nM. Cell cytotoxicity (B) was measured after 48 hours in the presence or absence of T cells. (C and D) BiTE-mediated induction of CD25 (C) and CD69 (D) cultured alone or in the presence of DLD-1 cells was measured by flow cytometry. (E and F) CD69 and CD25 were measured on CD4+ and CD8+ T cells by flow cytometry. (G) Percentage of interferon-gamma (IFN-γ) positive CD4+ and CD8+ T cells were measured after 6 hours in coculture with DLD-1 cells (5:1) and BiTE-containing supernatants. Degranulation of CD4+ and CD8+ T cells following addition of BiTE containing supernatants in coculture of DLD-1 and T cells was measured by CD107a externalization after 6 hours. Externalization was assessed by coculture with a CD107a-specific antibody followed by flow cytometry analysis (H). Secretion of granzyme B and perforin by BiTE-activated CD4+ and CD8+ T cells to mediate target cell killing by apoptosis was measured at 24 hours (I and J). (K) Cytokines released into supernatants were quantified by ELISA. Data show mean±SEM of biological triplicates. Statistical significance was assessed by two-way analysis of variance followed by Bonferroni post hoc analysis. Significance was assessed versus untreated cells within the relevant group (**p<0.01 and ***p<0.001). BiTE, bispecific T cell engager; IL, interleukin; PBMC, peripheral blood mononuclear cell; PD-L1, programmed death-ligand 1.

PD-L1-targeted BiTE was constructed by linking an scFv or nanobody (NB) recognizing human PD-L1^22 23^ to an scFv recognizing CD3ε (clone OKT3)^23^ via a flexible glycine-serine linker (G_4_-S)_3_. Control BiTEs were generated similarly and contained scFv or NB fragments targeting irrelevant antigens (scFv recognizing filamentous hemagglutinin adhesin of *Bordetella pertussis* 7 and an NB targeting rabies virus^8^). An N-terminal immunoglobulin signal sequence and C-terminal hexahistidine tag were added for mammalian secretion and detection (figure 1A and online supplemental figure 1A). Plasmid DNA encoding BiTE was transfected in HEK293A cells using Lipofectamine 2000 (Invitrogen, UK). Transfected cells were cultured for 48 hours, before supernatants were harvested and concentrated, as described previously.^7^ The BiTE concentration in the supernatant was quantified by anti-His Tag ELISA (GenScript, UK).

10.1136/jitc-2020-001292.supp1 Supplementary data

10.1136/jitc-2020-001292.supp2 Supplementary data

### Generation of oHSV-1 expressing BiTE

‘Armed’ oHSV-1 were constructed by insertion of the BiTE cassette into the parental oHSV-1 (G207 backbone cloned in a bacterial artificial chromosome (BAC))^21^ as described previously.^24^ BiTEs were placed under transcriptional control of the CMV promoter in the mutated ICP6 region. The modified BAC DNA was verified by Sanger sequencing (Eurofins Genomics, Germany) before virus rescue.^24^ A single viral plaque was amplified and concentrated by density-gradient centrifugation.^25^ Viral stocks were titred by Quant-iT Picogreen dsDNA assay (ThermoFisher Scientific) and agar overlay plaque assay.

### Processing of human peripheral blood mononuclear cells (PBMCs) and isolation of T cells

Human leukocyte cones from consenting healthy volunteers (NHS Blood and Transfusion Service, Oxford, UK) were used for PBMC isolation by density-gradient centrifugation using Ficoll-Paque Plus (GE Healthcare, UK). CD3+ cells were isolated using the Pan T-cell Isolation Kit (Miltenyi Biotech).

### Processing of clinical biopsy samples

Primary human malignant ascites samples were obtained from consented cancer patients at the Churchill Hospital (Oxford University Hospitals NHS Foundation Trust). Ascites samples were processed immediately, with cells and fluid separated by centrifugation as described previously.^26^ Online supplemental table 1 lists the ascites samples used in this study.

10.1136/jitc-2020-001292.supp3 Supplementary data

### Generation of monocyte-derived macrophages and polarization to M2-like phenotype

Monocytes were isolated from leukocyte cones by double density-gradient centrifugation.^8^ Monocytes were cultured in a low-adherence cell culture grade flask in X-VIVO 10 (Lonza, UK) medium supplemented with 2% (v/v) heat-inactivated human serum (Sigma-Aldrich, UK). On day 4, monocyte-derived macrophages were polarized to an M2 phenotype with interleukin (IL)-10 (10 ng/mL, Miltenyi Biotech, UK). Cells were harvested and plated for experiments 48 hours after polarization.

### T cell activation and target cell cytotoxicity assays

For PBMC-based experiments, CD3+ T cells were isolated and cocultured with DLD-1 human colorectal carcinoma cells at a ratio of 5:1. For *ex vivo* experiments, unpurified total ascites cells were seeded in normal serum (NS) medium (RPMI 1640, Sigma-Aldrich; supplemented with 2% FBS (v/v)) or autologous acellular ascites fluid mixed (50% v/v) with NS medium. Cultures were treated with free BiTEs (40 nM) or infected with virus. Where appropriate, CD3/CD28 Dynabeads (ThermoFisher Scientific, UK) were included as positive controls for T cell activation. T cells were harvested after 48 hours (free BiTE) or on day 4 (virus infection), and activation was assessed using fluorescently labeled antibodies against CD3, CD4, CD8, CD25 and CD69 (Biolegend, UK) using an Attune NxT Flow Cytometer (ThermoFisher Scientific, UK).

Cytotoxicity was measured by lactate dehydrogenase release (CytoTox 96 Non-Radioactive Cytotoxicity Assay; Promega), MTS viability assay (CellTiter 96 Cell Proliferation Assay, Promega) or in real-time by measuring cell impedance using xCELLigence (Acea Biosciences). Cell viability was determined by flow cytometry using a live-dead stain (Live/Dead Fixable Near-IR stain; Invitrogen, UK).

### Quantification of cytokines in supernatants

Cytokines and chemokines were measured by commercial ELISA kits (Biolegend, UK) or multiplex LEGENDplex Human Macrophage/Microglia panel kit (Biolegend, UK).

### Microscopy and live-cell imaging

M2 macrophages were stained with CellTrace Far-Red Cell Proliferation Kit (ThermoFisher Scientific) and cocultured 1:2 with tumor cells (DLD-1) in NS media. After 24 hours, cells were infected with parental or oHSV-1 expressing PD-L1 BiTEs at MOI 1. After 3 hours of infection, T cells stained with CellTrace Violet Cell Proliferation Kit (ThermoFisher Scientific) were added to the coculture. Uninfected cells were used as a negative control. Also, 2 µM NucView 530 Caspase-3 Substrate (Biotium) was added to each well to visualize apoptosis. Brightfield and fluorescence images were captured on a Nikon Ti-E Microscope fitted with Andor Zyla 4.2 sCMOS camera (10× optical objective) at intervals of 15 min for 4 days. Time-lapse movies (14 frames/s) were generated using Fiji software. Caspase-3 substrate signal was detected in Cy3 channel of fluorescence microscope (excitation/emission wavelength of 528/563 nm) and was undisturbed by viral EGFP cassette (FITC channel, excitation wavelength 488 nm). Green colour for Caspase was selected to display better contrast during image/movie processing using Fiji software (Figure 6D, Supplementary Figure 15 and Supplementary Movies 1-4).

### Statistical analysis

Statistical analyses were performed using two-way analysis of variance with Bonferroni post hoc analysis. All data are presented as mean±SEM. The significance levels used were p=0.01–0.05 (*), 0.001–0.01 (**) and 0.0001–0.001 (***). Experiments were performed in biological triplicate, unless otherwise stated.

## Results

### Generation and characterization of a BiTE targeting PD-L1

The ability of PD-L1 BiTEs to mediate activation of human lymphocytes and target cell toxicity was assessed by incubating them at increasing concentrations with PD-L1-positive DLD-1 cells (online supplemental figures 1B and 2) with or without human T cells. Whereas no cytotoxicity was observed in the absence of T cells, both anti-PD-L1 constructs triggered potent cytotoxicity against DLD-1, with nearly all target cells killed during the experiment (figure 1B). A significant increase in T cell activation markers CD25 and CD69 was observed in the presence of PD-L1 BiTEs (figure 1C, D). In the absence of target cells, the PD-L1 BiTEs caused a background level T cell activation, perhaps reflecting binding to PD-L1 on T cells or a level of aggregation of BiTEs allowing some crosslinking of CD3ε in the absence of target cells (figure 1C, D).

10.1136/jitc-2020-001292.supp4 Supplementary data

Interestingly, both CD4+ and CD8+ T cells demonstrated increased expression of CD69 and CD25 with significant increases in the generation of interferon-gamma (IFN-γ)-producing T cells (figure 1E–G and online supplemental figure 3). PD-L1 BiTEs cause degranulation of CD4+ and CD8+ T cells (figure 1H), mediating apoptosis of target cells (figure 1I, J and online supplemental figure 3B). Furthermore, we observed an increase in IFN-γ, IL-10 and IL-2 secretion in the presence of PD-L1 BiTEs, suggesting activation of both the Th1 and Th2 responses (figure 1K). No target cell killing, T cell activation, degranulation or cytokine release was observed with control BiTEs (figure 1B–K).

10.1136/jitc-2020-001292.supp5 Supplementary data

### PD-L1 BiTEs activate endogenous T cell cytotoxicity in unpurified human malignant ascites cell model

**Figure 2:**
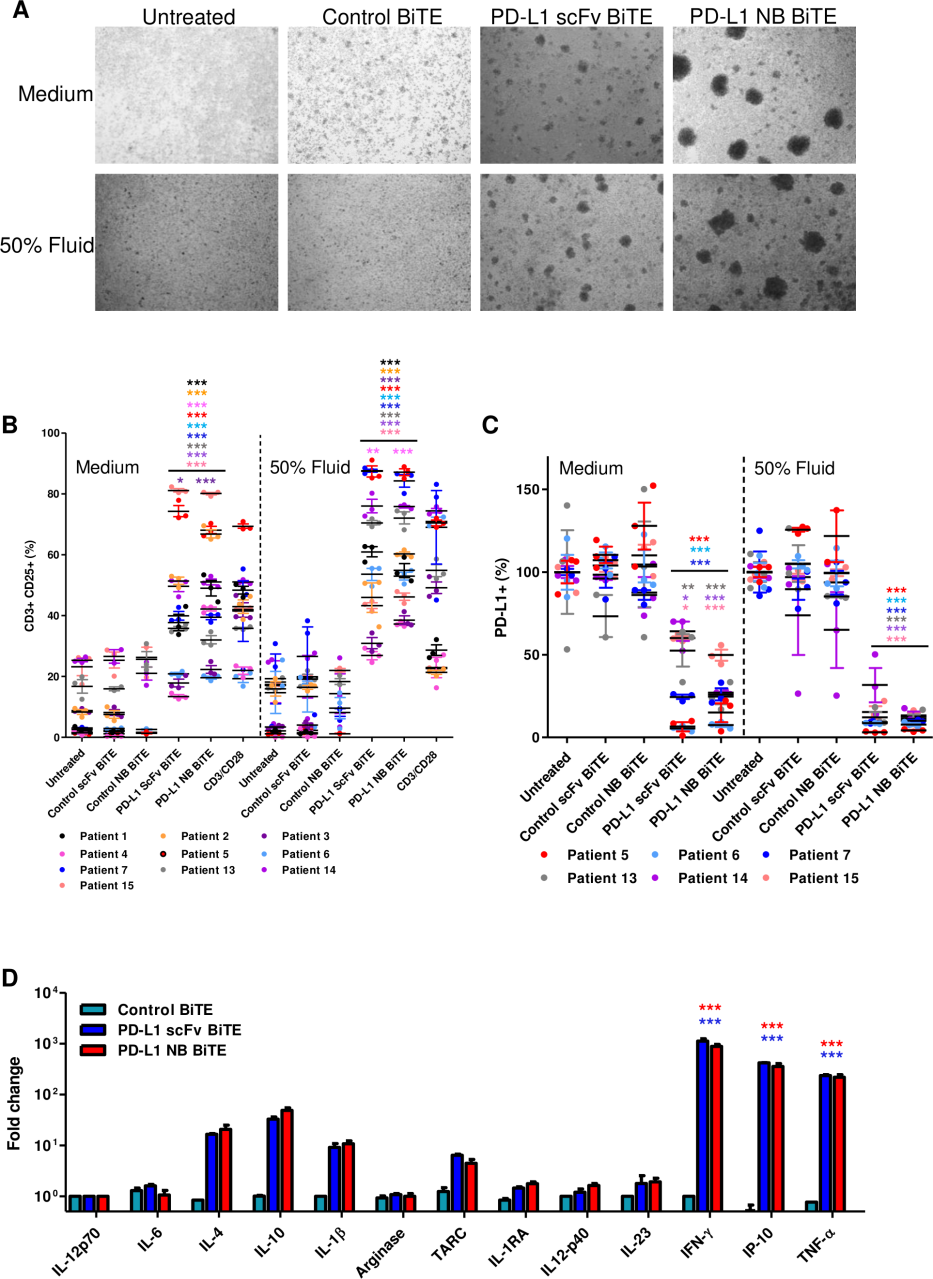
PD-L1 targeting BiTE activates endogenous ascites T cells to kill PD-L1-positive cells. (A) Representative brightfield images of unpurified ascites cells 48 hours after treatment with control scFv, PD-L1 scFv and PD-L1 NB BiTE in the presence of normal serum (NS) medium or autologous ascites fluid (50% v/v). (B and C) Total unpurified ascites cells from different patient samples were treated with PD-L1 BiTEs or their respective control for 72 hours in NS medium or autologous ascites fluid (50% v/v) before analysis by flow cytometry for CD25 expression on activated endogenous CD3+ T cells (B) or PD-L1-positive cells (C). (D) Cytokine release was evaluated after 72 hours by multiplex ELISA panel. Data show mean±SEM of biological triplicates. Statistical significance was assessed by two-way analysis of variance followed by Bonferroni post hoc analysis. Significance was assessed versus untreated cells within the relevant group (*p<0.05, **<0.01 and ***<0.001). BiTE, bispecific T cell engager; IFN-γ, interferon-gamma; IL, interleukin; NB, nanobody; PD-L1, programmed death-ligand 1; scFv, single-chain variable fragment.

Ascites is a pathological accumulation of fluid, usually in the peritoneal cavities of patients with ovarian, breast, gastrointestinal, pancreatic and uterine cancer.^26^ Ascites fluids are drained for palliation, and they often contain tumor cells, fibroblasts, myeloid cells and lymphocytes, making them an excellent cell model to assess BiTE efficacy in a fully controlled near-clinical environment (online supplemental figure 4). We harvested total cells from the ascites of patients with cancer (online supplemental tables 1 and 2) and incubated them with PD-L1 BiTEs and their relevant controls. The cells were suspended in NS medium or in the presence of autologous ascites fluid (50% v/v). After 48 hours, we observed cell clustering with PD-L1 BiTEs but not control BiTE (figure 2A), indicating T cell activation. Flow cytometry analysis after 72 hours showed high levels of endogenous T cell activation by the PD-L1 BiTEs, ranging between 13%–81.16% and 20%–80.06% CD25 positivity with PD-L1 scFv and PD-L1 NB BiTE, respectively (figure 2B and online supplemental figure 5). Both CD4+ and CD8+ T cells exhibited PD-L1 BiTE-induced expression of CD25 (online supplemental figure 6A, B). Greater activation of endogenous T cells was observed in the presence of autologous fluid (PD-L1 scFv 27%–88%, PD-L1 NB 37%–87%; figure 2B and online supplemental figure 5) compared with NS media. Furthermore, a robust depletion of PD-L1+ cells was observed following addition of PD-L1 BiTEs, which was more pronounced in the presence of ascites fluid (figure 2C and online supplemental figure 6C).

10.1136/jitc-2020-001292.supp6 Supplementary data

10.1136/jitc-2020-001292.supp7 Supplementary data

10.1136/jitc-2020-001292.supp8 Supplementary data

10.1136/jitc-2020-001292.supp9 Supplementary data

A multiplex immunoassay was used to assess the impact of BiTE treatment on cytokine release. Ascites cells (patient #7) were treated with PD-L1 BiTEs, and supernatants were harvested after 72 hours. Robust increases in IFN-γ (1000-fold), IP-10 (355-fold) and TNF-α (220-fold) levels were observed in samples treated with PD-L1 BiTEs compared with untreated samples. A strong fold increase in thymus and activation-regulated chemokine (TARC), IL-4, IL-10 and IL-1β was also observed (figure 2D). The high ratio values were partly due to low backgrounds measured in the absolute cytokine levels in untreated or control BiTE treated samples (online supplemental figure 7A).

10.1136/jitc-2020-001292.supp10 Supplementary data

These data indicate that PD-L1 scFv and PD-L1 NB BiTE mediate activation of endogenous T cells in ascites samples, triggering a dramatic shift in the inflammatory milieu.

### PD-L1 BiTE does not augment killing of activated T cells

**Figure 3:**
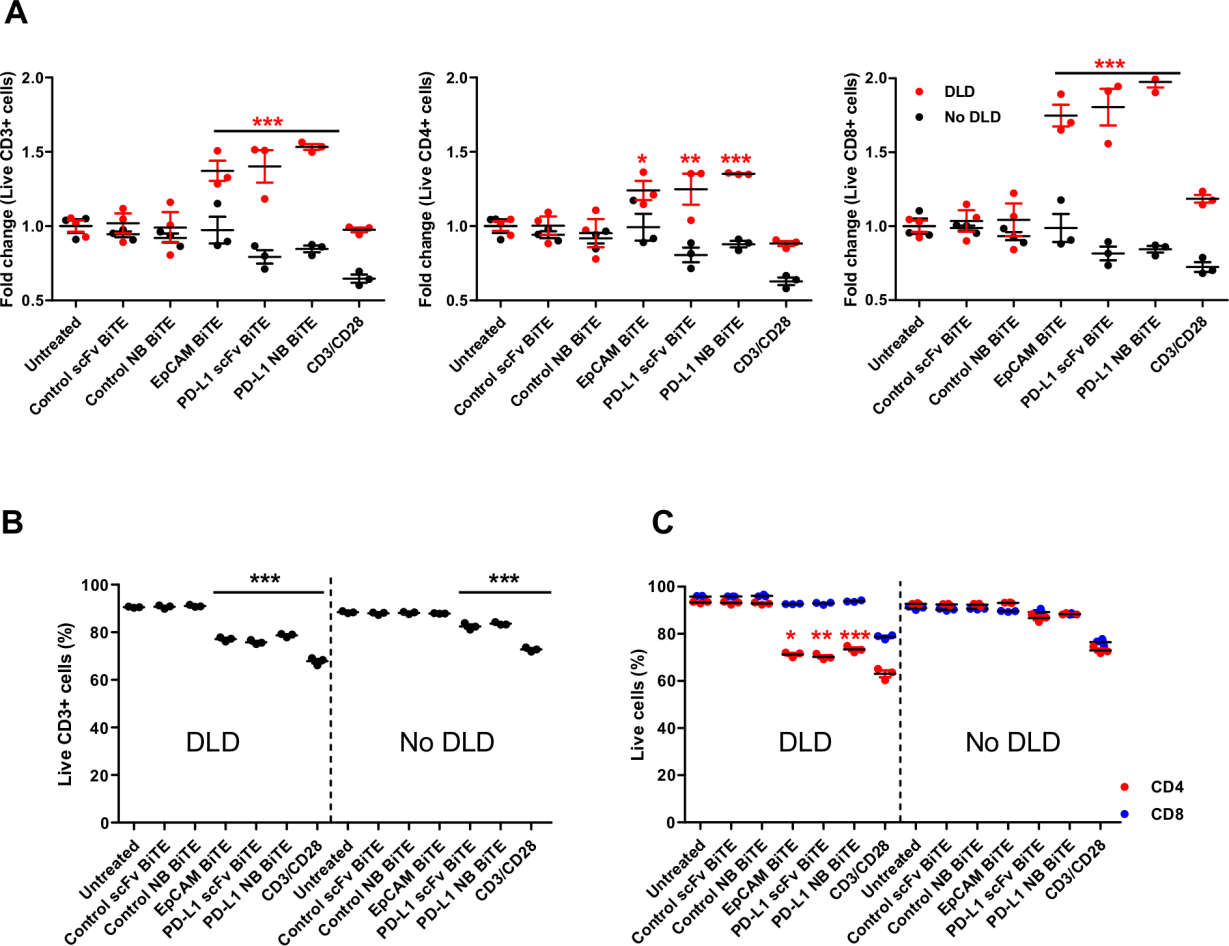
PD-L1 BiTE does not induce killing of activated T cells. PBMC-derived T cells were incubated with control-targeting, PD-L1-targeting or EpCAM-targeting BiTE and cocultured with DLD-1 carcinoma cells (5:1). T cells were harvested after 48 hours and analyzed by flow cytometry to calculate relative fold-change in live CD3+, CD4+ and CD8+ T cells (A). Total number of live CD3+ T cells (B) and CD4+ and CD8+ subsets (C) were calculated. Statistical significance was assessed by two-way analysis of variance followed by Bonferroni post hoc analysis. Significance was assessed versus untreated cells within the relevant group (*p<0.05, **p<0.01 and ***p<0.001). BiTE, bispecific T cell engager; PD-L1, programmed death-ligand 1.

Similar to previous studies, we observed PD-L1 expression on surface of activated T lymphocytes^27–29^ (online supplemental figures 4B, C and 7B). Therefore, bispecific engagers may target the effector cells to eliminate themselves in a process known as fratricide. We examined T cell killing in a coculture with tumor cells (DLD-1), control BiTEs or PD-L1 BiTEs. CD3/CD28 Dynabeads and an EpCAM BiTE, which targets EpCAM on tumor cells and CD3ε on T cells,^7^ were included as positive controls for activation-induced cell death (AICD) and BiTE-induced T cell death. An increase in total T cell numbers was observed in every sample treated with EpCAM or PD-L1 BiTEs in the presence of DLD-1 cells, thought to reflect T cell proliferation following formation of an activational synapse. The increase in number of CD8+ cells was always greater than the increase in CD4+ cells, with numbers of CD8+ cells more than doubling compared with DLD-1-free controls (figure 3A). No significant T cell expansion was observed with the control BiTEs. Interestingly, T cell proliferation was lower with the CD3/CD28 beads than the synapse-forming BiTEs (figure 3A). We assessed whether fratricide was occurring despite increased total T cell numbers. In the presence of DLD-1 cells and PD-L1 BiTEs, we observed an 11%–15% decrease in the proportion of live CD3+ T cells (untreated: 90%, PD-L1 scFv BiTE: 75%, PD-L1 NB BiTE: 78%; figure 3B). A similar decrease (13%) in the total percentage of live CD3+ cells was observed with EpCAM BiTE, suggesting that the effect is a product of BiTE-mediated activation, and not a PD-L1-specific effect. There was no decrease in viability of live CD3+ cells with control BiTEs, suggesting that the effect was dependent on CD3 clustering, most likely AICD. CD3+ cells treated with CD3/CD28 Dynabeads experienced a greater decrease in viability (23% lower than the untreated well; figure 3B). Interestingly, CD4+ T cells were more susceptible to AICD than CD8+ T cells, with 19%, 23% and 20% fewer live CD4+ T cells in EpCAM, PD-L1 scFv and PD-L1 NB BiTE, respectively (figure 3C).

### Immunosuppressive ascites fluid increases PD-L1 expression on tumor cells

**Figure 4:**
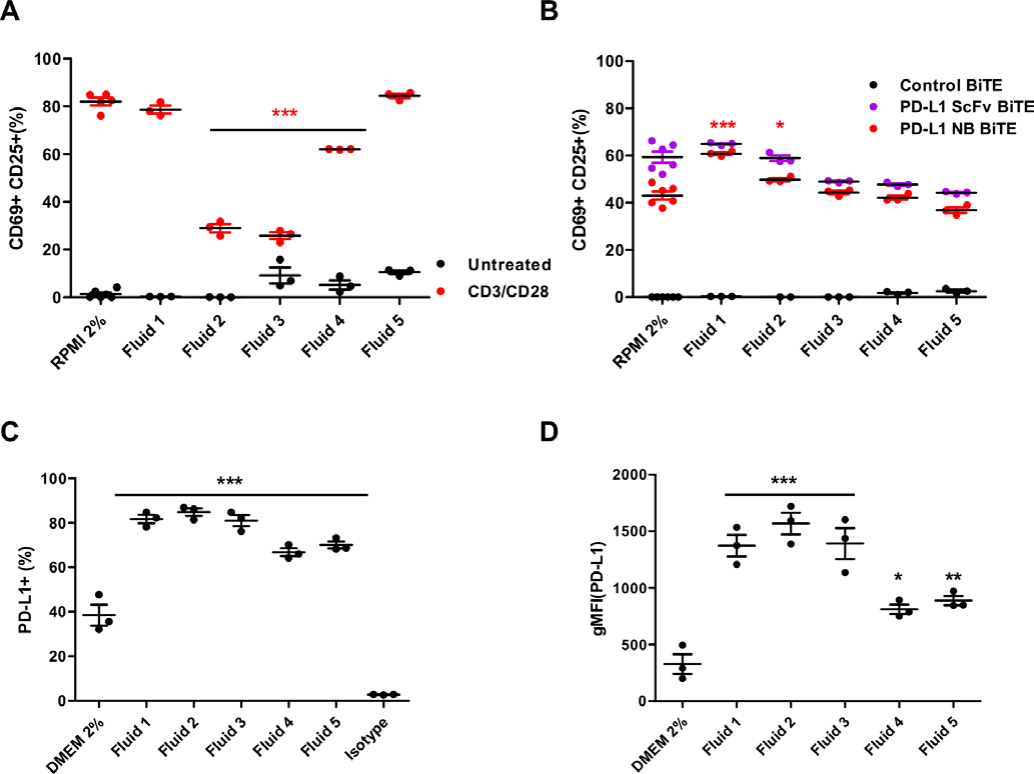
Effect of immunosuppressive ascites fluids on PD-L1-targeting BiTE and tumor cells. (A) PBMC-derived T cells were incubated with CD3/CD28 Dynabeads in normal serum (NS) medium or patient-derived malignant ascites fluids (50% v/v) for 48 hours before measuring for T cell activation (CD69+/CD25+) by flow cytometry. (B) PBMC-derived T cells were cocultured with DLD-1 carcinoma cells (5:1) and incubated with either PD-L1 scFv or PD-L1 NB BiTE in the presence of NS medium or patient-derived ascites fluids (50% v/v) for 48 hours before measuring for CD69+/CD25+ expression by flow cytometry. (C and D) DLD-1 cells were grown in NS medium or 50% (v/v) patient-derived ascites fluid for 48 hours before analysis by flow cytometry for the PD-L1-positive population (C) and geometric mean fluorescence intensity (D). Statistical significance was assessed by two-way analysis of variance followed by Bonferroni post hoc analysis. Significance was assessed versus untreated cells within the relevant group (*p<0.05, **p<0.01 and ***p<0.001). BiTE, bispecific T cell engager; DMEM, Dulbecco’s modified Eagle medium; NB, nanobody; PD-L1, programmed death-ligand 1; PBMC, peripheral blood mononuclear cell; scFv, single-chain variable fragment.

Malignant ascites fluids contain various soluble factors (cytokines, chemokines and growth factors) that inhibit immune activation and accentuate an immunosuppressive environment while providing a supportive environment for cell growth and tumor spread.^26 30^ These fluids recapitulate many features of the TME and provide an excellent translational model system. To characterize the effect of ascites fluid on T cell activation, we stimulated PBMC-derived T cells with CD3/CD28 Dynabeads in NS medium and in the presence of different ascites fluids (50% v/v). Marked activation of T cells was observed in NS medium, with approximately 60%–80% T cells positive for both CD69 and CD25 (figure 4A and online supplemental figure 7C). A range of suppression was observed with different ascites fluids. While some do not appear to influence T cell activation (fluid 1–78% CD69+CD25+, fluid 5–84% CD69+CD25+; figure 4A), fluids 2 and 3 strongly inhibit activation (23% CD69+CD25+) and fluid 4 does so moderately (65% CD69+CD25+; figure 4A).

In contrast, there was no suppressive effect of any ascites fluid on PD-L1 scFv or PD-L1 NB BiTE activity. Robust T cell activation was achieved in all tested fluids (figure 4B), even in those shown to suppress activation by CD3/CD28 beads. Interestingly, activation in ascites fluids was equal to or greater than that in NS medium. Next, we assessed if increased activation of T cell in the presence of ascites fluid was specific for PD-L1 BiTE by measuring T cell activation induced by EpCAM BiTE in the presence of ascites fluids compared with PD-L1 BiTE. We observed approximately 10% higher T cell activation with EpCAM BiTE than PD-L1 BiTE in NS medium but significantly decreased activation in four out of five tested ascites fluids (online supplemental figure 7D). These results confirm that PD-L1 BiTEs can bypass ascites fluid-associated T cell immunosuppression.

We also assessed the impact of ascites fluids on PD-L1 expression in target cells. DLD-1 cells were cultured in the presence of NS media or ascites fluid. After 48 hours, cells were harvested and stained for PD-L1 expression. We observed a significant increase in the percentage of PD-L1-positive cells (up to 40%; figure 4C) and geometric mean fluorescence intensity (gMFI) value (from 2.5-fold to 5-fold increase; figure 4D) in the presence of ascites fluid. The effect was notably higher for strongly immunosuppressive fluids (fluids 2 and 3). These observations suggest that ascites fluids increase PD-L1 expression on tumor cells, which may provide more targets for the PD-L1 BiTE, explaining the greater efficacy of the PD-L1 BiTE in some ascites fluids.

### Expression of PD-L1 BiTE from oHSV-1

**Figure 5:**
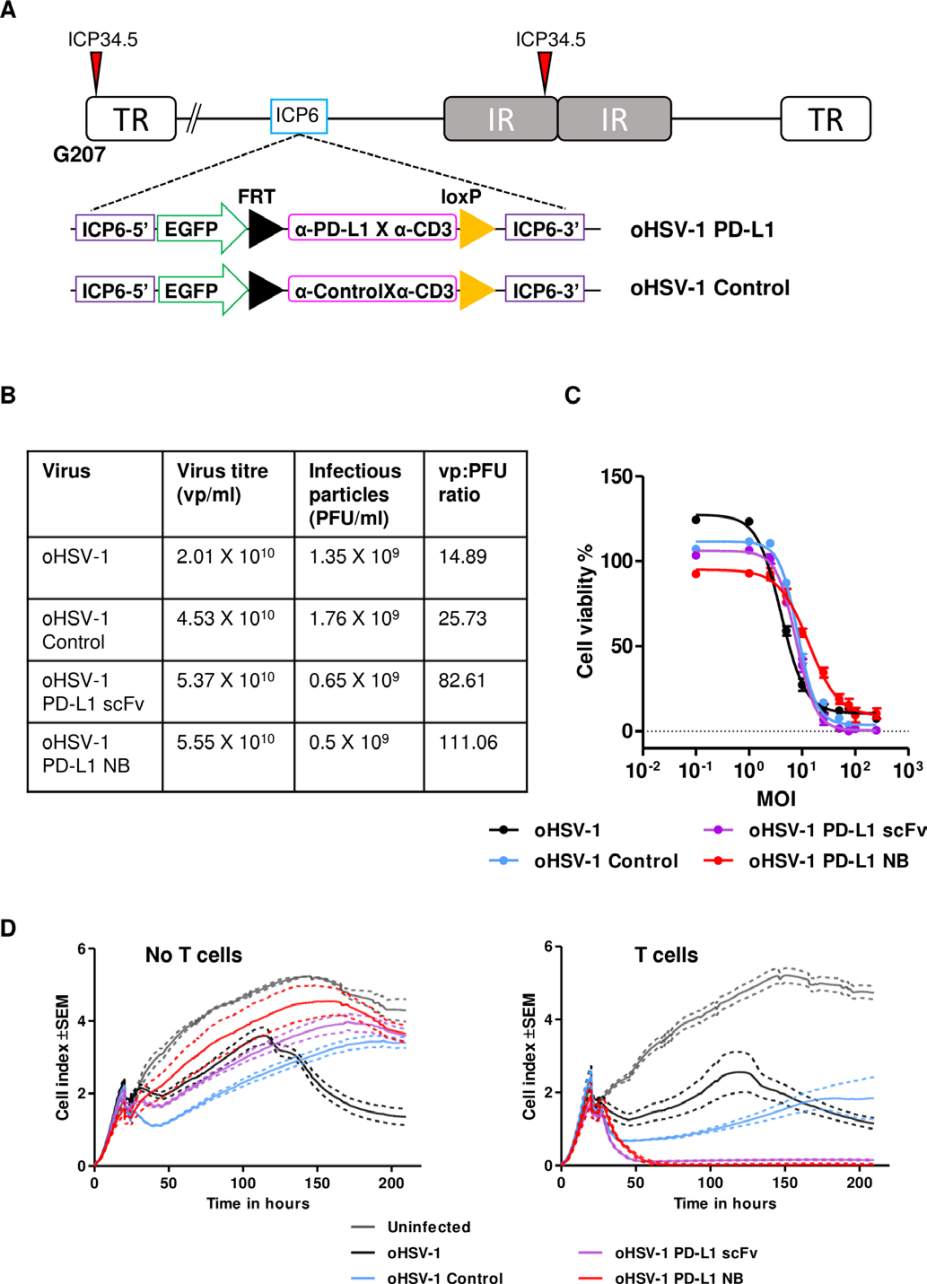
Generation of oncolytic herpes simplex virus-1 (oHSV-1) expressing PD-L1 targeting BiTE. (A) Schematic representation of the oHSV-1 genome used in the study. G207 is a second-generation oHSV-1 with deletions in both copies of infected cell protein 34.5 (ICP34.5) and an inactivating insertion into ICP6. A transgene expressing PD-L1 BiTE or a non-specific control BiTE was inserted into the backbone of G207 at ICP6 locus using the FLP-FRT recombination. (B) Table outlining the virus titer and infectious particle number of parental and each recombinant BiTE expressing oHSV-1, determined by dsDNA Picogreen assay and plaque agar assay, respectively. Particle-to-infectivity ratios for parental virus and each BiTE armed virus was calculated. (C) DLD-1 cells were incubated with serial dilutions of parental or oHSV-1 expressing PD-L1 or control BiTE. Five days after infection, cell viability was determined by MTS assay. (D) Cytotoxicity of armed oHSV-1 was monitored in real-time over 200 hours using xCELLigence. DLD-1 cells were seeded and infected with parental oHSV-1 or BiTE-armed oHSV-1 in the absence or presence of T cells (1:5). Impedance was measured at 15 min intervals. Data represent three biological triplicates, with means represented by a solid line and the SEM with dotted line. TR, terminal repeat; IR, internal repeat; PFU, plaque-forming unit; MOI, multiplicity of infection; BiTE, bispecific T cell engager; NB, nanobody; PD-L1, programmed death-ligand 1; scFv, single-chain variable fragment.

G207 is an oHSV-1 with a deletion in the major neurovirulence gene ICP34.5 and a mutation in the ICP6 ribonucleotide reductase gene, enabling it to replicate selectively in and kill cancer cells.^3 21^ Clinical trials using G207 have demonstrated virus potency and safety in patients with cancer^31–33^ that can be further potentiated by encoding biologicals for expression within tumor cells.^34^ We engineered oHSV-1 to express PD-L1 scFv, PD-L1 NB and control BiTE (see Materials and Methods; figure 5A). Viruses were rescued, and genome replication and particle-to-infectivity ratios were assessed (figure 5B and online supplemental figure 8). The approximate quantity of active PD-L1 BiTE produced from oHSV-1 infected cells was determined by comparing cytotoxicity induced by viral infected supernatants to cytotoxicity induced by known quantities of recombinant PD-L1 BiTE (online supplemental figure 9A, B). Compared with parental oHSV-1, we observed slight attenuation in oncolytic potency of recombinant oHSV-1 (figure 5C) due to transgene insertion.

10.1136/jitc-2020-001292.supp11 Supplementary data

10.1136/jitc-2020-001292.supp12 Supplementary data

We then evaluated the potency of BiTE secreted from virus-infected cells in a coculture of T cells and tumor cells. Robust T cell activation and tumor cell cytotoxicity was observed using diluted supernatant (1:100) from cells infected with oHSV-1 expressing PD-L1 BiTEs but not from cultures infected with oHSV-1 expressing control BiTE or the parental oHSV-1 (online supplemental figure 9C, D). We observed T cell-mediated reduction in the viability of target DLD-1 cells with parental and control viruses. To validate this observation, we repeated the experiment with T cells isolated from PBMCs of two different donors. We observed a small increase in the early (CD69) but not late (CD25) T cell activation confirming the reduction in the viability was due to non-specific activation of T cells when cocultured with oHSV-1 and tumor cells (online supplemental figure 9E).

Next, we monitored oncolysis mediated by BiTE-armed oHSV-1 in real-time using xCELLigence. DLD-1 cells were infected with parental and BiTE-armed oHSV-1 (MOI 1), then 3 hours later, human PBMC-derived T cells were added. In the absence of T cells, no cytotoxicity of target cells was observed with any of the BiTE-armed viruses (figure 5D) except for the parental oHSV-1, for which cytotoxicity was observed after 75 hours (figure 5D). The decrease observed in oncolysis was MOI-dependent as, at increased MOI (MOI 10) BiTE-armed viruses also exhibited cell cytotoxicity (online supplemental figure 10). In the presence of T cells, rapid cell lysis was observed within 16 hours of infection by oHSV-1 expressing PD-L1 BiTE, with complete cytotoxicity at 24 hours (figure 5D). No cytotoxicity was observed with oHSV-1 expressing control BiTE in the presence of T cells.

10.1136/jitc-2020-001292.supp13 Supplementary data

### oHSV-1 expressing PD-L1 BiTE can overcome immune-suppressive ascites fluids and are toxic to both tumor cells and polarised M2-like macrophages

**Figure 6:**
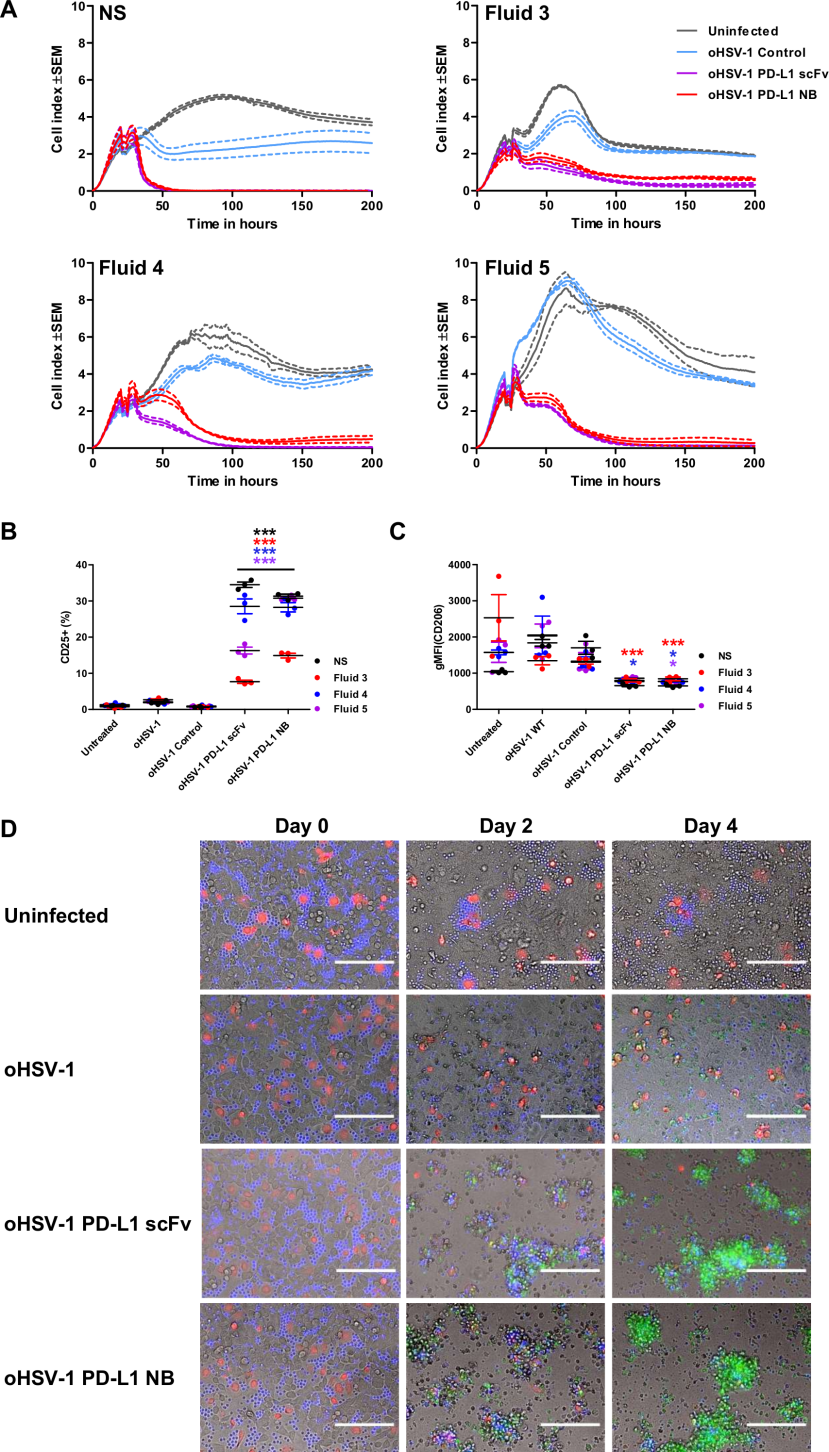
oHSV-1 expressing PD-L1 BiTE can overcome immune-suppressive effects of ascites fluid and mediate killing of tumor cells and M2-like macrophages. (A) Cytotoxicity of BiTE-expressing oHSV-1 was monitored at 15 min intervals over 200 hours using xCELLigence. DLD-1 cells were infected with parental oHSV-1 or BiTE expressing oHSV-1 at an MOI of 1. PBMC-derived T cells (5:1) were added 3 hours postinfection in the presence of normal serum (NS) medium or ascites fluids (50% v/v). (B and C) Monocyte-derived macrophages from healthy donor PBMCs were polarized to M2-like macrophages using IL-10. After 48 hours of polarization, cells were infected with parental oHSV-1 or BiTE expressing oHSV-1 at MOI 1. Autologous T cells (1:5) were added 3 hours postinfection in the presence of NS medium or ascites fluids (50% v/v). Cells were analyzed by flow cytometry after 5 days for expression of CD25 (B) and CD206 (C). (D) Representative images of cocultures of DLD-1 carcinoma cells (unlabelled), polarized M2-like macrophages (red) and autologous CD3+ T cells (blue), infected with parental oHSV-1, oHSV-1 expressing PD-L1 scFv, PD-L1 NB BiTE or uninfected. Apoptosis was visualized using caspase-3 substrate (green). Scale bar: 1 mm. Full time-lapse sequences are displayed in online supplemental movies S1–S4. Statistical significance was assessed by two-way analysis of variance followed by Bonferroni post hoc analysis. Significance was assessed versus untreated cells within the relevant group (*p<0.05 and ***p<0.001). BiTE, bispecific T cell engager; NB, nanobody; oHSV-1, oncolytic herpes simplex virus-1; PD-L1, programmed death-ligand 1; scFv, single-chain variable fragment.

We measured the replication kinetics of the viruses and secretion of PD-L1 BiTE in the presence of malignant ascites fluids (online supplemental figure 11). In parallel, the ability of oHSV-1-delivered PD-L1 BiTE to drive T cell activation was assessed. DLD-1 cells were infected with oHSV-1 expressing control and PD-L1 BiTEs at MOI 1. PBMC-derived T cells (1:5) and ascites fluids (50% v/v) were added 3 hours after infection. We selected fluids 3, 4 and 5 to model high, moderate and low immunosuppression, respectively (figure 4A). We observed a distinct increase in cell index when cells were cultured in the presence of ascites fluids compared with NS media (5.5–8.5 vs 4), suggesting that growth factors in ascites fluid promote proliferation of tumor cells (figure 6A). Target cell cytotoxicity was observed with PD-L1 BiTE-armed viruses, but not control virus, in all fluids (figure 6A), confirming that BiTEs expressed via oHSV-1 can activate T cells and mediate target cell killing, even in the presence of immunosuppressive ascites fluids. PD-L1 BiTE-mediated killing in the presence of highly immunosuppressive fluid 3 was not noticeably different from killing in the other fluids. However, the time required to achieve 100% cytotoxicity was increased threefold in all samples of ascites fluid compared with NS media (75 hours vs 24 hours, respectively). This may reflect the increased growth of the target cancer cells in the presence of ascites, although it might also indicate decreased herpes virus infection due to the presence of complement in the ascites samples. Next, we assessed if effect of ascites fluid on oHSV-1 infection can be overcome at higher virus concentrations. We observed MOI-dependent cytotoxic efficiency of ‘armed’ oHSV-1 with 100% cytotoxicity obtained at MOI 10 (online supplemental figure 12).

10.1136/jitc-2020-001292.supp14 Supplementary data

10.1136/jitc-2020-001292.supp15 Supplementary data

10.1136/jitc-2020-001292.supp16 Supplementary video

10.1136/jitc-2020-001292.supp17 Supplementary video

10.1136/jitc-2020-001292.supp18 Supplementary video

10.1136/jitc-2020-001292.supp19 Supplementary video

Previous studies have shown that TAMs promote cancer cell proliferation, invasion, metastasis and production of immune-suppressive molecules.^35^ In particular, TAMs exhibiting an M2-like phenotype are directly involved in T cell suppression by expressing surface PD-L1.^35^ To determine whether PD-L1 BiTE can target T cells to TAMs, we differentiated human monocytes to macrophages and polarized them to an M2-like phenotype. M2-like macrophages were 40% CD206+ and nearly 100% PD-L1+ (online supplemental figure 13A). They were infected with parental or BiTE-armed oHSV-1 in the presence of NS media or malignant ascites fluids. oHSV-1 infected macrophages expressed PD-L1 BiTE and triggered significant expansion and activation of PBMC-derived CD3+ cells (CD25+ up to 34%; figure 6B and online supplemental figure 13B) in NS and all ascites fluids tested, while parental or control BiTE-expressing oHSV-1 remained ineffective. T cell activation driven by PD-L1 scFv BiTE, however, was attenuated in fluids 3 and 5 (CD25+ 7% and 18%, respectively) compared with fluid 4 and NS, whereas PD-L1 NB BiTE maintained T cell activation similar to NS media in fluids 4 and 5 and showed attenuation with fluid 3 (CD25+ 15%; figure 6B). CD206 is a marker of TAMs and correlated as an inhibitor of antitumor immune response^36^ and a poor prognostic marker of cancer.^37^ We observed a significantly lower level of CD206 in surviving cells after polarized M2-like macrophages were treated with PD-L1 BiTEs both in NS media and in the presence of ascites fluids (figure 6C).

10.1136/jitc-2020-001292.supp20 Supplementary data

Next, we tested whether PD-L1 BiTE mediates simultaneous cytotoxicity of tumor cells and TAMs. Cocultures of tumor cells (DLD-1, unlabelled), human PBMC-derived M2-like macrophages (red) and T cells (blue) were infected with parental oHSV-1 or PD-L1 BiTE-armed oHSV-1. Cultures were monitored for 4 days using time-lapse microscopy (online supplemental movies S1–S4). Dramatic cytotoxicity was observed towards both tumor cells and M2-like macrophages in wells treated with oHSV-1 encoding scFv or NB PD-L1 BiTEs, with complete apoptosis of both cell types on day 4, as evidenced by the green caspase stain (figure 6D). We observed both tumor cells (EpCAM+) and M2 macrophages (CD11b+) positive for oHSV-1 (GFP+), producing active PD-L1 BiTE (online supplemental figures 11B–D and 14A) and mediating significant increase in number of CD3+ cells but decrease in tumor and M2-like macrophages (online supplemental figure 14B). We also observed complete apoptosis in M2-like macrophages infected with ‘armed’ oHSV-1, confirming PD-L1 BiTE-mediated targeting of TAMs (online supplemental figure 15).

10.1136/jitc-2020-001292.supp22 Supplementary data

10.1136/jitc-2020-001292.supp23 Supplementary data

### oHSV-1 expressing BiTE activate endogenous T cells in patient-derived malignant ascites model and kills different cell types

**Figure 7:**
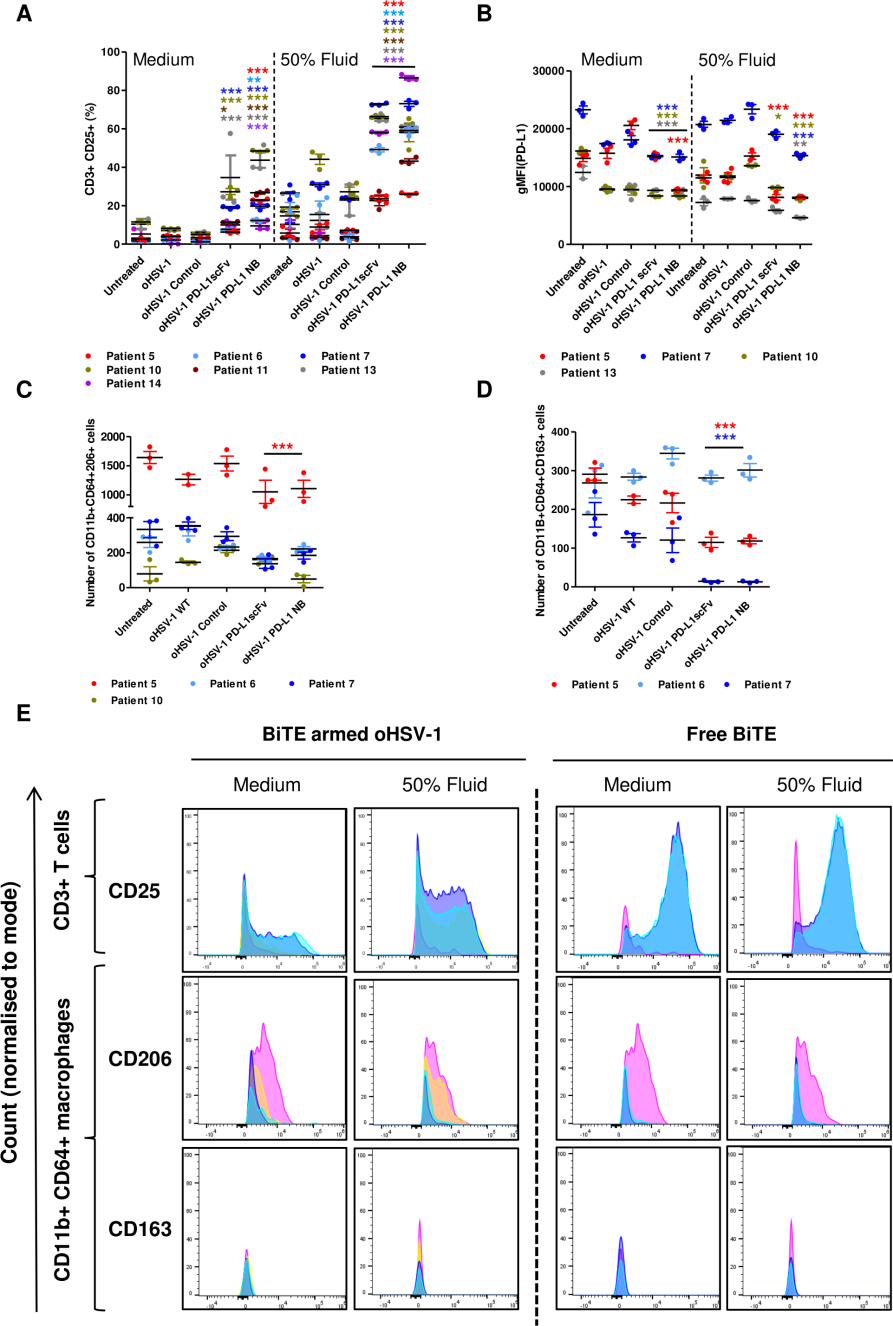
oHSV-1 expressing PD-L1 BiTEs activates endogenous ascites T cells to kill ascites tumor cells and macrophages. Total unpurified ascites cells from different patients were infected with parental oHSV-1 or BiTE expressing oHSV-1 at an MOI of 1 for 4 days in normal serum medium or in the presence of autologous fluid (50% v/v). Endogenous T cell activation (CD3+ CD25+) (A) and PD-L1 expression (B) were determined by flow cytometry. Cytotoxicity of CD206+ (C) and CD163+ (D) ascites macrophages in the presence of autologous ascites fluid (50% v/v) were analyzed by staining in the CD11b+/CD64+ population. (E.) Histograms of surface CD25 expression on endogenous ascites T cells (CD3+) and CD206 and CD163 expression on ascites macrophages (CD11b+/CD64+) infected with parental oHSV-1 (yellow), BiTE expressing oHSV-1 or incubated with free PD-L1 BiTEs (40 nM). Untreated: pink, PD-L1 scFv: blue, PD-L1 NB: cyan. Statistical significance was assessed by two-way analysis of variance followed by Bonferroni post hoc analysis. Significance was assessed versus untreated cells within the relevant group (*p<0.05, **p<0.01 and ***p<0.001). BiTE, bispecific T cell engager; NB, nanobody; oHSV-1, oncolytic herpes simplex virus-1; PD-L1, programmed death-ligand 1; scFv, single-chain variable fragment

We next tested the cytotoxic activity of virus-encoded PD-L1 BiTE in a human malignant ascites-based cell model. Whole malignant ascites cells were infected for 4 days with recombinant or parental oHSV-1 (MOI 1) in NS media or in the presence of autologous ascites fluid (50% v/v). PD-L1 BiTE-armed oHSV-1 triggered activation of endogenous T cells as measured by significant induction of CD25 (PD-L1 scFv: 6%–34.6%, PD-L1 NB: 12%–48%). CD25 induction was markedly higher in the presence of autologous ascites fluids for most patients than in NS medium (PD-L1 - scFv: 23%–72%, PD-L1 NB: 26%–86.5%; figure 7A and online supplemental figure 16). Interestingly, in three out of four patient samples, we observed a decrease in the level of PD-L1 expression on oHSV-1-treated cancer cells, although this effect was not seen in the presence of ascites fluid (figure 7B). Ascites samples treated with oHSV-1-encoding PD-L1 BiTE showed a lower level of PD-L1 expression on surviving cells, indicating selective cytotoxicity toward PD-L1-positive cells or downregulation of PD-L1 expression. This decrease was more pronounced in the presence of ascites fluid, again fitting with the possibility that immunosuppressive components of the fluid may augment this therapeutic approach (figure 7B).

10.1136/jitc-2020-001292.supp24 Supplementary data

We then examined TAMs in ascites samples infected with PD-L1 BiTE-armed oHSV-1 in the presence of autologous ascites fluid and observed a decreased number of CD11b+CD64+CD206+ and CD11b+CD64+CD163+ cells relative to untreated samples (figure 7C, D). Flow cytometry spectra assessing the expression levels of CD25 by endogenous CD3+ T cells as well as CD206 and CD163 expressed by CD11b+CD64+ ascites macrophages in PD-L1 BiTEs treated samples (both as free BiTE and viral-encoded protein; as quantified in figures 2B and 7A, C, D) are displayed in figure 7E. Untreated or parental oHSV-1 treated samples were used to display control expression levels. A more detailed flow cytometric analysis of different cell populations was performed on one patient sample (patient #5) treated with free BiTE. There was a significant decrease in the number of tumor cells (EpCAM+, PD-L1+) and M2 like macrophages (CD11b+CD64+CD206+ and CD11b+CD64+CD163+) with PD-L1 BiTE treatment, alongside a significant increase in activated T cells number (CD3+ and CD25+) (online supplemental figure 17 and 18).

10.1136/jitc-2020-001292.supp25 Supplementary data

## Discussion

Here we have developed a strategy to broaden the cytotoxic activity of an oncolytic herpesvirus, oHSV-1, by encoding within it a PD-L1-specific BiTE for secretion by infected cancer cells. This approach seeks to combine direct oncolysis of malignant cells with inhibition of paracrine and immunological support from non-transformed cells. PD-L1 was chosen as a suitable pan-cancer marker since it is widely expressed within tumors, not only on tumor cells, but also present on immunosuppressive cells, such as TAMs, which are important mediators of antitumor immune inhibition and tumor progression.^18 38 39^ We have shown that the PD-L1 BiTE mediates T cell activation and antigen-specific cytotoxicity with the release of cytotoxic granules for destruction of target cells.^40^ Inserting a BiTE expression cassette into oHSV-1 genome restricts BiTE-mediated T cell cytotoxicity to the tumor, maximizing anticancer effects while minimizing ‘on-target off-tumor’ systemic toxicities.

While PD-L1 is expressed on several disease-supporting cell types within the tumor, it is also expressed on the surface of T cells themselves.^27 29^ PD-L1 ligation has been linked with T cell-mediated expression of IL-10,^28^ although PD-L1-positive tumor-infiltrating lymphocytes (TILs) do appear functional, as they express IFN-γ and CD107a.^41^ A recent study revealed that PD-L1+ T cells engage with PD-1+ macrophages and induce differentiation of suppressive M2-like macrophages.^42^ Here PD-L1-positivity did not prevent the effector function of BiTE-activated T cells as significant target cytotoxicity, and release of proinflammatory cytokines was achieved independent of PD-L1 status (figures 1 and 2). Despite their expression of PD-L1, we saw no reduction in the number of live T cells in the presence of PD-L1 BiTE, suggesting either that PD-L1 on T cells is not accessible for BiTE binding or that T cells do not mediate significant fratricide following PD-L1 cross-linking (figure 3). Precisely how T cells avoid cytotoxicity is unclear, although it is established that T cells express high levels of granzyme inhibitors^43^ and may thereby resist granzyme-mediated cytotoxicity from other T cells. Under the same conditions, we observed complete destruction of tumor cells and polarized M2-like macrophages when infected separately (online supplemental figure 15) or in a coculture (figure 6D) with oHSV-1 expressing PD-L1 BiTE.

Patient-derived peritoneal malignant ascites provides a clinically relevant model for assessment of emerging cancer therapeutics, enabling mechanistic insights into PD-L1 BiTE efficacy in a heterogeneous TME with associated immunosuppression. This model avoids involvement of murine stromal cells that compromise many *in vivo* model systems.^44^ Previous studies characterized lymphocytes in ascites (tumour-associated lymphocytes, TALs) to be more similar to tumor (TILs)^45^ than blood in displaying exhaustion markers (LAG-3, PD-1 and TIM-3).^46^ We observed significant activation of tumor-associated T cells with both PD-L1 BiTEs when added as free protein or expressed via an oncolytic viral vector (figures 2B and 7A). An increase in T cell activation was observed alongside a significant decrease in ascites tumor cells and macrophages (PD-L1+ and CD206+/CD163+ population; figures 2C and 7B–D). We also observed proinflammatory modification of the TME, with significant release of cytokines and chemokines, including IFN-γ, IP-10 and TNF-α (figure 2D).

We observed that the PD-L1 BiTE could mediate significant activation of both CD4+ and CD8+ T cell subsets, in line with the concept that most T cells (even those expressing PD-L1, data not shown) can be repurposed for BiTE-mediated cytotoxicity.^7^ The immunosuppressive effects of ascites fluids on lymphocytes are well established.^47^ Nevertheless, BiTE-mediated activation of endogenous T cells was maintained in the presence of immunosuppressive malignant ascites fluids (figure 4A, B), suggesting that BiTE-mediated activation may overcome local immunosuppressive effects.

Unexpectedly, the activation of endogenous T cells by PD-L1 BiTE, present as free protein or expressed by oHSV-1, was seen to be greater in the presence of autologous ascites fluid compared with NS media. This was coupled with the observation that the presence of ascites fluids led to significantly greater PD-L1 expression on the surface of tumor cells (figure 4C, D). This, in turn, is thought to provide more target epitopes for PD-L1 BiTE, thereby enhancing PD-L1 BiTE-mediated T cell activation via greater CD3 clustering or engaging more T cells for killing. Supporting this concept, we observed greater activation of ascites-associated T cells and destruction of TAMs (identified as being CD11b+CD64+CD206+ and CD11b+CD64+CD163+) and downregulation of PD-L1 in the presence of autologous ascites fluids (figure 7). In this way, BiTEs targeting PD-L1 may be able to subvert the immunosuppressive tumor environment to potentiate immunotherapy.

In our study, we compared the scFv and NB versions of the PD-L1 BiTE *in vitro* and *ex vivo* to determine any differences in T cell activation or tumor targeting. ScFv comprise two variable domains (VL and VH), while NB is composed of a single VH domain. Compared with scFv, NBs are smaller, have superior solubility and stability at pH extremes and achieve excellent tissue penetration *in vivo*.^48^ Occasionally, bispecific constructs exhibit poor potency, possibly due to a lack of clustering or engaging epitopes that are too far from the cell surface or due to the presence of bulky antigens. A tight synapse is more likely achieved with smaller nanobodies. Nevertheless, in this study, we observed similar results with both scFv and NB forms of PD-L1 BiTE in terms of T cell activation, proinflammatory cytokine release and cytotoxicity toward PD-L1-positive target cells.

## Conclusions

Arming oHSV-1 with PD-L1 BiTE can target cytotoxicity toward tumor cells but also kills TAMs, critical contributors to tumor progression and immune suppression. The BiTE-mediated cytotoxicity was able to overcome immunosuppressive ascites fluids, and cytotoxicity was augmented, not inhibited, by the immunosuppressive milieu. We believe the mechanism involves upregulation of PD-L1 expression on target cells within ascites, facilitating improved activation of endogenous T cells leading to greater target-mediated cytotoxicity. To our knowledge, this concept has not been demonstrated before and represents a simple approach to exploit immune-suppressive tumor escape strategies and converting them to opportunities for targeted immunotherapy.

10.1136/jitc-2020-001292.supp21 Supplementary data

## Data Availability

Data are available on reasonable request.
