# Supplementary material for: Oncolytic herpesvirus expressing PD-L1 BiTE for cancer therapy: exploiting tumor immune suppression as an opportunity for targeted immunotherapy
Source: J Immunother Cancer. 2021 Apr 5;9(4):e001292. doi: 10.1136/jitc-2020-001292 (PMC8026026; doi:10.1136/jitc-2020-001292)
Supplement: Supplementary data [file jitc-2020-001292supp001.pdf]

Supplementary Figure 1

PD-L1 expression on DLD-1 carcinoma cells. A. HEK293A cells were transfected with plasmid expressing PD-L1 BiTEs or control BiTEs. Supernatants were harvested 48 hours after and analysed for BiTE expression by western blotting using anti-His primary antibody and an HRP-conjugated anti-mouse secondary antibody. **B.** Level of PD-L1 expressed on the surface of human colorectal carcinoma cells DLD-1 was measured by flow cytometry (cyan). Unstained cells (red) were used to determine background fluorescence.

Supplementary Figure 2

Dose-response curves of PD-L1 BiTEs. PBMC-derived T cells were incubated with increasing concentrations of PD-L1 scFv or PD-L1 NB BiTE and co-cultured with DLD-1 carcinoma cells (5:1). A decrease in the viability of target cells after 48 hours was measured by MTS assay **(A)**. **B, C.** Activation of PBMC-derived CD3+ T cells was analysed by flow cytometry to calculate surface expression of CD25 **(B)** or CD69 **(C).** EC50 values are displayed in each plot.

Supplementary Figure 3

T cell activation by the PD-L1-targeting BiTEs. Healthy human PBMC-derived T cells were incubated with control or PD-L1 BiTEs in the presence of DLD-1 cells (5:1). Activation of CD4+ or CD8+ T cells was assessed through flow cytometric measurement of CD69 and CD25 **(A)**. **B.** Flow cytometry plots displaying interferon-gamma (IFN-γ)-producing, degranulating (CD107a) and granzyme B and perforin secreting CD4+ and CD8+ T cells.

Supplementary Figure 4

Representative example of the screening of malignant ascites cells to determine cellular composition by flow cytometry. A. Flow cytometry dot plots demonstrating the gating strategy used to assess cellular composition of ascites sample (Patient #13). Data were acquired with an Attune NxT flow cytometer then processed with FlowJo software. Cell doublets in total acquired cells (top row, left panel) were excluded by plotting FSC-area(A) against FSC-height(H) (top row,

middle panel). Total live cells were gated on singlet population using Live/Dead Fixable stain (top row, right panel). Cell types present in the ascites sample were determined by antibody staining for PD-L1 (macrophages, tumour), EpCAM (tumour), CD11b (myeloid) and CD3 (T cell) (bottom row panels). This example is non-exhaustive, and not all stains and gating strategies are displayed. Other markers included in characterisation panel are- FAP (fibroblast), CD4 (T cell), CD8 (T cell), CD56 (NK cell) CD206 (tumour associated macrophage), CD163 (tumour associated macrophage) and PD-1 (T cell exhaustion). Cellular composition of all human malignant ascites samples used in this study is listed in Supplementary Table-2. **B, C.** Representative flow cytometry plots showing lymphocytes, tumour cells and myeloid population positive for PD-L1; CD3+/PD-L1+, EpCAM+/PD-L1+ and CD11b+/PD-L1+ cells (red square). Percentage of dual positive population in different patient-derived ascites sample is listed in the table **(C)**. NA - Data not available.

Supplementary Figure 5

PD-L1 targeting BiTE-mediated activation of endogenous ascites T cells. Ten patient-derived samples of unpurified ascites cells were treated with PD-L1 scFv, PD-L1 NB and their respective control BiTEs in the presence of normal serum medium or autologous ascites fluid (50% v/v). 72 hours later, total cells were analysed by flow cytometry for CD25 expression on activated endogenous CD3+ T cells **(A)**. The analysis for each patient sample is provided as an individual plot. **B.** Representative flow cytometry plots of five ascites samples (Patient 5, 6, 7, 13 and 14) showing CD3+ CD25+ T cell population. Statistical significance was assessed by two-way ANOVA followed by Bonferroni post hoc analysis. Significance was assessed versus untreated cells within the relevant group (*, $P < 0.05$, **, $P < 0.01$ and ***, $P < 0.001$).

Supplementary Figure 6

PD-L1 targeting BiTEs activate both CD4+ and CD8+ subsets of endogenous ascites T cells and kill PD-L1-positive cells. Total unpurified ascites cells from different patient samples were treated with PD-L1 scFv, NB and their respective controls BiTEs for 72 hours in normal serum medium or autologous ascites fluid (50% v/v). Cells were analysed by flow cytometry for CD25

expression on CD4+ and CD8+ T cells (**A**). **B.** Representative flow cytometry plots showing activation (CD25+) of endogenous CD4+ and CD8+ T cells of a malignant ascites sample (Patient 12). **C.** Patient samples of unpurified ascites cells treated with PD-L1 scFv, PD-L1 NB and their respective control BiTEs were analysed by flow cytometry for PD-L1-positive cells. Statistical significance was assessed by two-way ANOVA followed by Bonferroni post hoc analysis. Significance was assessed versus untreated cells within the relevant group (*, $P < 0.05$, **, $P < 0.01$ and ***, $P < 0.001$).

Supplementary Figure 7

PD-L1 BiTEs induce inflammatory cytokine release and display enhanced T cells activation in the presence of immunosuppressive ascites fluids. The absolute cytokine release by BiTE treated or untreated endogenous ascites T cells was evaluated after 72 hours by multiplex ELISA panel (**A**). **B.** PD-L1 expression on surface of resting (left) and activated T cells (activated by CD3/CD28 Dynabeads; right) was measured by flow cytometry. **C.** PBMC-derived T cells were incubated with CD3/CD28 Dynabeads in normal serum (NS) media or patient-derived malignant ascites fluids (50% v/v) for 48 hours before measuring for T cell activation (CD25+) by flow cytometry. **D.** PBMC-derived T cells were co-cultured with DLD-1 carcinoma cells (5:1) and incubated with EpCAM BiTE, PD-L1 scFv BiTE or control BiTE at a dose of 40 nM in the presence of NS medium or patient-derived ascites fluids (50% v/v). After 48 hours, T cells were analysed by flow cytometry to calculate surface expression of CD25. Statistical significance was assessed by two-way ANOVA followed by Bonferroni post hoc analysis. Significance was assessed versus untreated cells within the relevant group (*, $P < 0.05$, ***, $P < 0.001$).

Supplementary Figure 8

***In vitro* assessment of genome replication of oHSV-1 encoding BiTE.** Human colorectal carcinoma cell line DLD-1 were infected with parental or BiTE encoding recombinant oHSV-1 at an MOI of 1 and 10. Cells were harvested, and supernatant were collected at 24, 48 and 72 hours post-infection. Genome replication was assessed by using qPCR primers and probes directed to the

HSV-1 DNA polymerase (UL42) gene fraction. The nucleic acid sequence of the forward primer was 5’-GCCAGCGAGACGCTGAT-3’, the reverse primer was 5’-ACGCAGGTACTCGTGGTGA-3,’ and the fluorescent HSV-1 typing probe was 5’-CGCGAACTGACGAGCTTTGTGGT-3’. Each condition was measured in biological triplicate and represented as mean ± SEM.

Supplementary Figure 9

Quantification of PD-L1 BiTE secreted from virus-infected DLD-1 cells. A. Standard curve of lactate dehydrogenase (LDH) released (Abs490) into supernatants after 48 hours co-culture of PBMC-derived T cells and DLD-1 carcinoma cells (5:1) with varying quantities of recombinant PD-L1 scFv or PD-L1 NB BiTE. **B.** DLD-1 cells were infected with parental or BiTE expressing oHSV-1 and supernatants were harvested 72 hours after the infection. In parallel, fresh DLD-1 and T cell co-culture wells were made and incubated with harvested diluted viral supernatants. LDH release was measured 48 hours post-incubation with the indicated viral supernatant. The dilution used to determine the quantity of PD-L1 BiTEs is indicated with a tick. A concentration of approximately 43.27 μM and 32.14 μM PD-L1 scFv and PD-L1 NB BiTE respectively were produced per 0.1 million of infected DLD-1 cells over 72 hours in 500 μl medium with normal serum. Each condition was measured in biological triplicate and represented as mean ± SEM. **C.** A co-culture with DLD-1 and PBMC-derived T cells were incubated with diluted supernatants (1:100) harvested from Vero cells infected at an MOI of 1. After 48 hours, target cell viability was assessed by MTS assay and T cell activation (CD25+) was measured by flow cytometry (**D**). **E.** Target cell (DLD-1) viability and T cell activation were measured (as described in **C** and **D**) but with T cells-derived from PBMCs of two different human donors. Statistical significance was assessed by two-way ANOVA followed by Bonferroni post hoc analysis. Significance was assessed versus untreated cells within the relevant group (*, $P < 0.05$, ***, $P < 0.001$).

Supplementary Figure 10

oHSV-1 expressing PD-L1-targeting BiTE induces MOI-dependent cytotoxicity of tumour cells. Cytotoxicity of parental and BiTE armed oHSV-1 was monitored in real-time using xCELLigence. DLD-1 carcinoma cells were seeded and infected with parental or BiTE-armed oHSV-1 at MOI of 1 and 10. The cytotoxicity was measured over 280 hours. Data represents three biological triplicates, with means represented by a solid line.

Supplementary Figure 11

***In vitro* assessment of genome replication and BiTE production by recombinant oHSV-1 in the presence of immunosuppressive ascites fluid. A.** Human colorectal carcinoma cells DLD-1 cells were infected with parental or BiTE encoding recombinant oHSV-1 at MOI 1 and 10 in the presence of ascites fluid (Fluid 6, 50% v/v). Cells were harvested, and supernatant were collected at 24, 48 and 72 hours post-infection. Genome replication was assessed by using qPCR primers and probes directed to the HSV-1 DNA polymerase (UL42) gene fraction. The nucleic acid sequence of the forward primer was 5'-GCCAGCGAGACGCTGAT-3', the reverse primer was 5'-ACGCAGGTACTCGTGGTGA-3', and the fluorescent HSV-1 typing probe was 5'-CGCGAACTGACGAGCTTTGTGGT-3'. **B, C.** Standard curve of lactate dehydrogenase (LDH) released (Abs490) into supernatants after 48 hours co-culture of PBMC-derived T cells and DLD-1 (5:1) with varying quantities of recombinant PD-L1 scFv **(B)** or PD-L1 NB BiTE **(C)**. **D.** DLD-1 cells were infected with parental or BiTE expressing oHSV-1 in normal serum (NS) medium, in the presence of ascites fluid (Fluid 6, 50%v/v) or co-cultured with human monocyte-derived macrophages polarised to M2-like phenotype (2:1). Supernatants were harvested 72 hours after the infection and added to fresh DLD-1 and T cell co-cultures wells in different dilutions. LDH release was measured 48 hours post-incubation with the indicated viral supernatant. The dilution used to determine the quantity of PD-L1 BiTEs is indicated with a tick. PD-L1 scFv BiTE was secreted at a level of approximately 43.27 µM, 39.30 µM and 39.51 µM (per 500 µl, 72 hours) per 0.1 million of infected DLD-1 cells in NS media, ascites fluid and M2 macrophages respectively. PD-L1 NB BiTE was produced at 32.14 µM, 32.17 µM and 27.71 µM per 0.1 million DLD-1 cells when

infected in NS media, malignant ascites fluids and in co-culture with M2 macrophages respectively. Each condition was measured in biological triplicate and represented as mean ± SEM.

Supplementary Figure 12

PD-L1 BiTE expressed from oHSV-1 induces unhindered T cell-mediated lysis of tumour cells despite the presence of immune-suppressive ascites fluids. Viability of human carcinoma cell line DLD-1 was monitored in real-time over 220 hours by an xCELLigence-based cytotoxicity assay. DLD-1 cells were cultured with human PBMC-derived T cells (1:5) and infected with parental or BiTE expressing oHSV-1 at MOI 1 **(A, B)** and 10 **(C, D)**. Patient-derived malignant ascites fluid (Fluid 6) was added (50% v/v) at the time of infection **(B, D)** or 3 hours post-infection **(A, C)**. Impedance was measured at 15-minute intervals. Data represents three biological triplicates, with means represented by a solid line and the SEM with dotted line.

Supplementary Figure 13

oHSV-1 expressing PD-L1 BiTE mediated activation of T cells in coculture with polarised M2-like macrophages. A. Monocyte-derived macrophages from healthy donor PBMCs were polarised to M2-like macrophages using IL-10 for 48 hours. Surface PD-L1 and CD206 were measured on polarised M2 and unpolarised macrophages (M0) by flow cytometry. **B.** Polarised M2 macrophages were infected with parental or BiTE encoding recombinant oHSV-1 at an MOI 1 and co-cultured with autologous CD3+ T cells (1:5). Cells were harvested after 72 hours, and relative fold-change in live CD3+ T cells was calculated by flow cytometry. Polarised M2-like macrophages were infected with parental or BiTE armed oHSV-1 in the presence of normal serum (NS) media or three different ascites fluids (50% v/v; Fluid 3, 4 and 5). **C.** Representative flow cytometry plots showing activation (CD25+) of autologous CD3+ T cell.

Supplementary Figure 14

PD-L1 BiTE expressed from oHSV-1 mediate T cell-dependent killing of tumour cells and human PBMC-derived M2-like macrophages. A. A co-culture of human colorectal carcinoma

cell line DLD-1 and healthy human PBMC-derived macrophages polarised to M2-like phenotype (1:1) was infected with parental or BiTE armed oHSV-1. 72 hours after infection, cells were harvested, and percentage of each cell type (DLD-1 - EpCAM+ and M2-like macrophages - CD11b+) positive for oHSV-1 (GFP+) was calculated by flow cytometry. **B.** PBMC-derived T cells were co-cultured with DLD-1 carcinoma cells and autologous M2- like macrophages and infected with parental or BiTE expressing oHSV-1 for 72 hours. Fold-change in live cells were calculated with flow cytometric analysis after staining with anti-CD3, anti-EpCAM, anti-CD11b and a fixable Live/Dead stain. Statistical significance was assessed by two-way ANOVA followed by Bonferroni post hoc analysis. Significance was assessed versus untreated cells within the relevant group (*, $P < 0.05$, ***, $P < 0.001$).

Supplementary Figure 15

oHSV-1 expressing PD-L1 targeting BiTE induces T-cell-dependent cytotoxicity of M2-like macrophages. A. Monocyte-derived macrophages from healthy donor PBMCs were polarised to M2-like macrophages using IL-10 for 48 hours. Polarised M2 macrophages were infected with parental or BiTE encoding recombinant oHSV-1 at an MOI 1 and co-cultured with autologous CD3+ T cells (1:5). Representative images showing polarised M2-like macrophages (red) and autologous CD3+ T cells (blue), infected with parental oHSV-1, oHSV-1 expressing PD-L1 scFv, PD-L1 NB BiTE or uninfected. Apoptosis was visualised using Caspase-3 Substrate (green). Scale bar, 1 mm.

Supplementary Figure 16

oHSV-1 expressing PD-L1 BiTEs mediated activation of endogenous ascites T cells. Total unpurified ascites cells from different patients were infected with parental oHSV-1 or BiTE expressing oHSV-1 at an MOI of 1 for four days in normal serum medium or in the presence of autologous fluid (50% v/v). Endogenous T cell activation (CD3+/CD25+) **(A)** were determined by flow cytometry. The analysis for each patient sample is provided as an individual plot. **B.** Representative flow cytometry plots of five ascites samples (Patient 5, 6, 10, 13 and 14) showing

CD3+ CD25+ T cell population. Statistical significance was assessed by two-way ANOVA followed by Bonferroni post hoc analysis. Significance was assessed versus untreated cells within the relevant group (*, P < 0.05, **, P < 0.01 and ***, P < 0.001).

Supplementary Figure 17

Representative gating strategies to determine different cell population in unpurified malignant ascites sample. Patient-derived malignant ascites cells treated with PD-L1 BiTE and its relevant control or untreated were harvested and stained with anti-CD3, anti-CD25, anti-PD-L1, anti-EpCAM, anti-CD11b, anti-CD64, anti-CD163 and anti-CD206 antibodies, or relevant isotype controls. Representative gating is displayed to determine positivity for the indicated marker. Data were acquired with an Attune NxT flow cytometer then processed with FlowJo software. Cell doublets were excluded by plotting FSC-area(A) against FSC-height(H) (top left panel). Within this gate, CD3-, PD-L1-, EpCAM-positive cells were selected (middle panels) and analysed. Gating for CD25-positive cells was made on CD3-positive cells (top right panel). Single cells were gated for CD11b- and CD64-positive cells to select macrophages (CD11b+, CD64+, bottom left panel). M2-like macrophages were selected on CD11b+, CD64+ cells and gated for CD163- and CD206-positive population. Representative plots display cell population from an untreated well.

Supplementary Figure 18

PD-L1 BiTE activates T cells to kill different cell types in *ex vivo* model. Total unpurified ascites cells from patient number 5 were treated with PD-L1 scFv or PD-L1 NB BiTE (40 nM) for 72 hours before analysis by flow cytometry. Representative plots of different cell population are shown. Percentages for different cell populations are given in the table.

Supplementary Table-1

A list of patients-derived malignant ascites samples used in this study.

Supplementary Table-2

Cellular composition of the patient-derived malignant ascites samples used in this study.

Supplementary Movie S1

Time-lapse sequence of uninfected culture. Time-lapse sequences showing co-cultures of human colorectal carcinoma cell line DLD-1 (unlabelled), human PBMC-derived macrophages polarised to M2-like phenotype (red) and autologous CD3+ T cells (blue). Apoptosis was visualised using NucView 530 Caspase-3 substrate (green). Images were collected at intervals of 15 minutes covering for four days; original magnification 10X.

Supplementary Movie S2

Time-lapse sequence of co-culture infected with parental oHSV-1. Time-lapse sequences showing co-cultures of human colorectal carcinoma cell line DLD-1 (unlabelled), human PBMC-derived macrophages polarised to M2-like phenotype (red) and autologous CD3+ T cells (blue) infected with parental oHSV-1 (MOI 1). Apoptosis was visualised using NucView 530 Caspase-3 substrate (green). Images were collected at intervals of 15 minutes covering for four days; original magnification 10X.

Supplementary Movie S3

Time-lapse sequence of co-culture infected with oHSV-1 expressing PD-L1 scFv. Time-lapse sequences showing co-cultures of human colorectal carcinoma cell line DLD-1 (unlabelled), human PBMC-derived macrophages polarised to M2-like phenotype (red) and autologous CD3+ T cells (blue) infected with PD-L1 scFv BiTE armed oHSV-1 (MOI 1). Apoptosis was visualised using NucView 530 Caspase-3 substrate (green). Images were collected at intervals of 15 minutes covering for four days; original magnification 10X.

Supplementary Movie S4

Time-lapse sequence of co-culture infected with oHSV-1 expressing PD-L1 NB. Time-lapse sequences showing co-cultures of human colorectal carcinoma cell line DLD-1 (unlabelled), human PBMC-derived macrophages polarised to M2-like phenotype (red) and autologous CD3+ T cells (blue) infected with oHSV-1 expressing PD-L1 NB BiTE (MOI 1). Apoptosis was visualised using

NucView 530 Caspase-3 substrate (green). Images were collected at intervals of 15 minutes covering for four days; original magnification 10X.
