# Supplementary figures and images for: Oncolytic herpesvirus expressing PD-L1 BiTE for cancer therapy: exploiting tumor immune suppression as an opportunity for targeted immunotherapy
Source: J Immunother Cancer. 2021 Apr 5;9(4):e001292. doi: 10.1136/jitc-2020-001292 (PMC8026026; doi:10.1136/jitc-2020-001292)

## Supplementary Figure 1

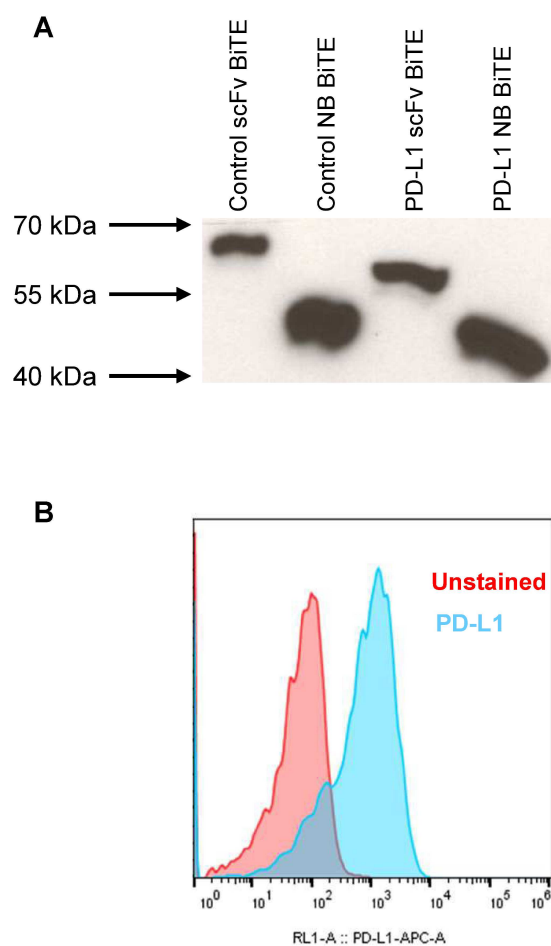

Supplement: Supplementary data [file jitc-2020-001292supp002.pdf]

## Supplementary Figure 2

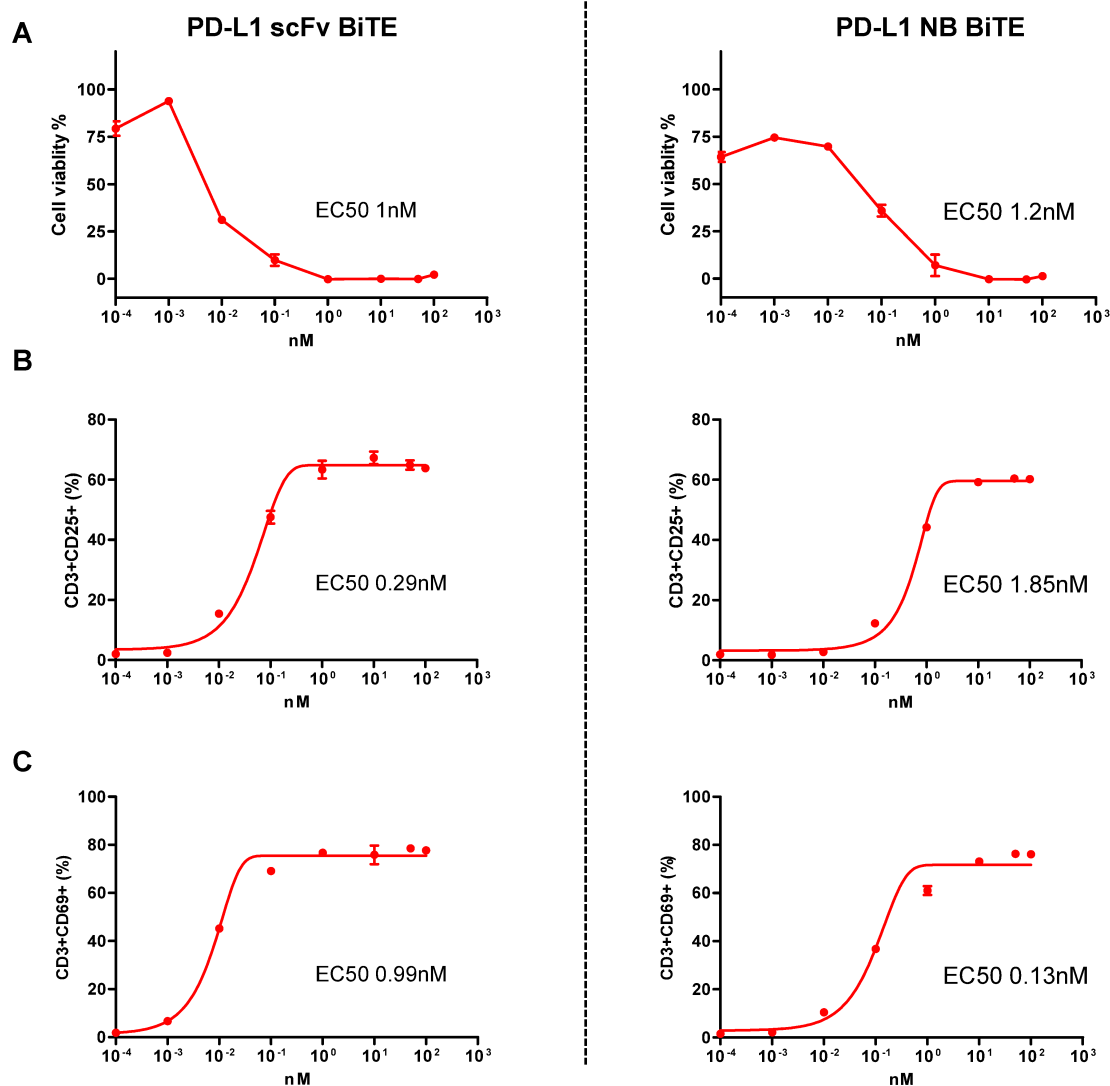

Supplement: Supplementary data [file jitc-2020-001292supp004.pdf]

Supplementary Figure 3

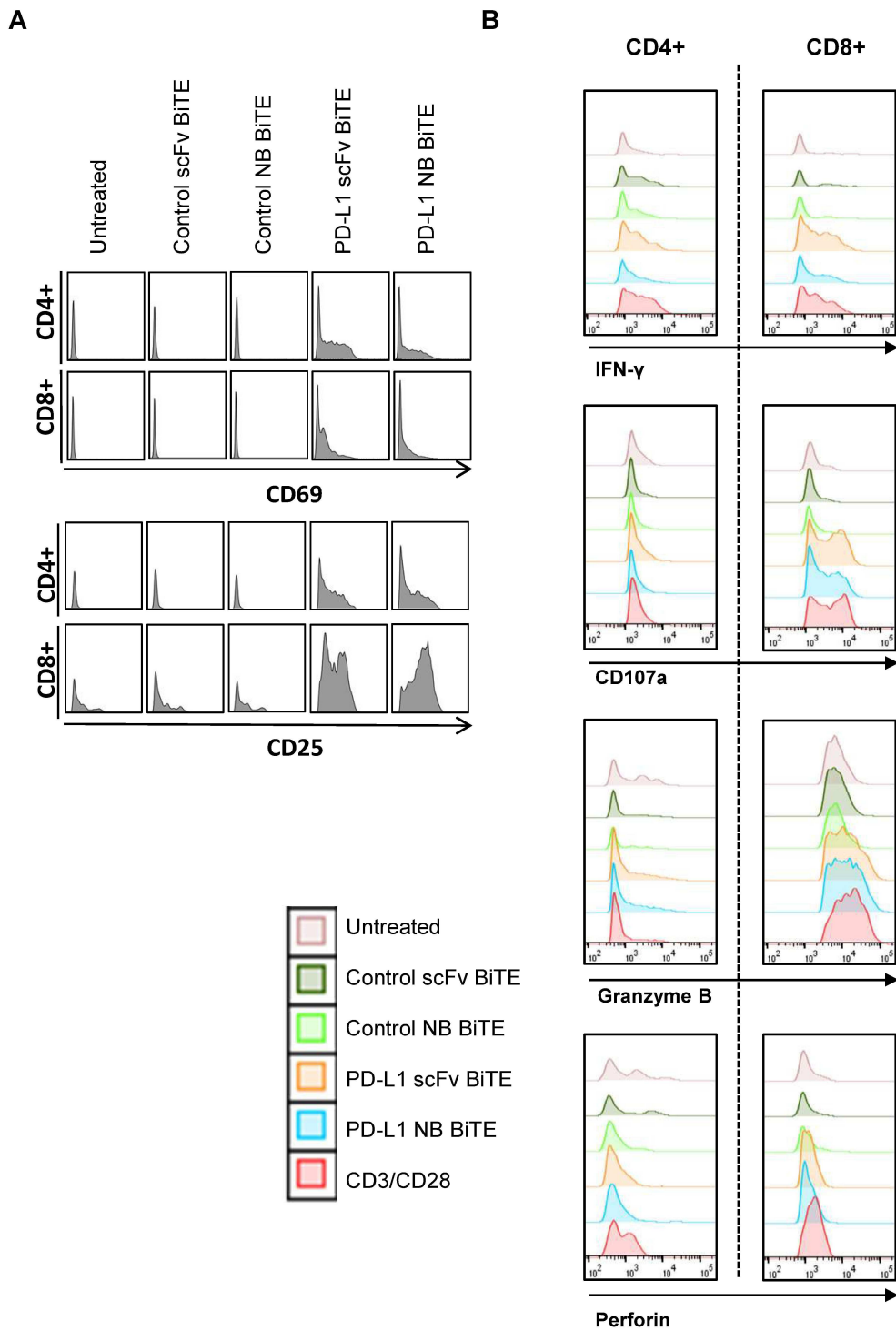

Supplement: Supplementary data [file jitc-2020-001292supp005.pdf]

Supplementary Figure 4

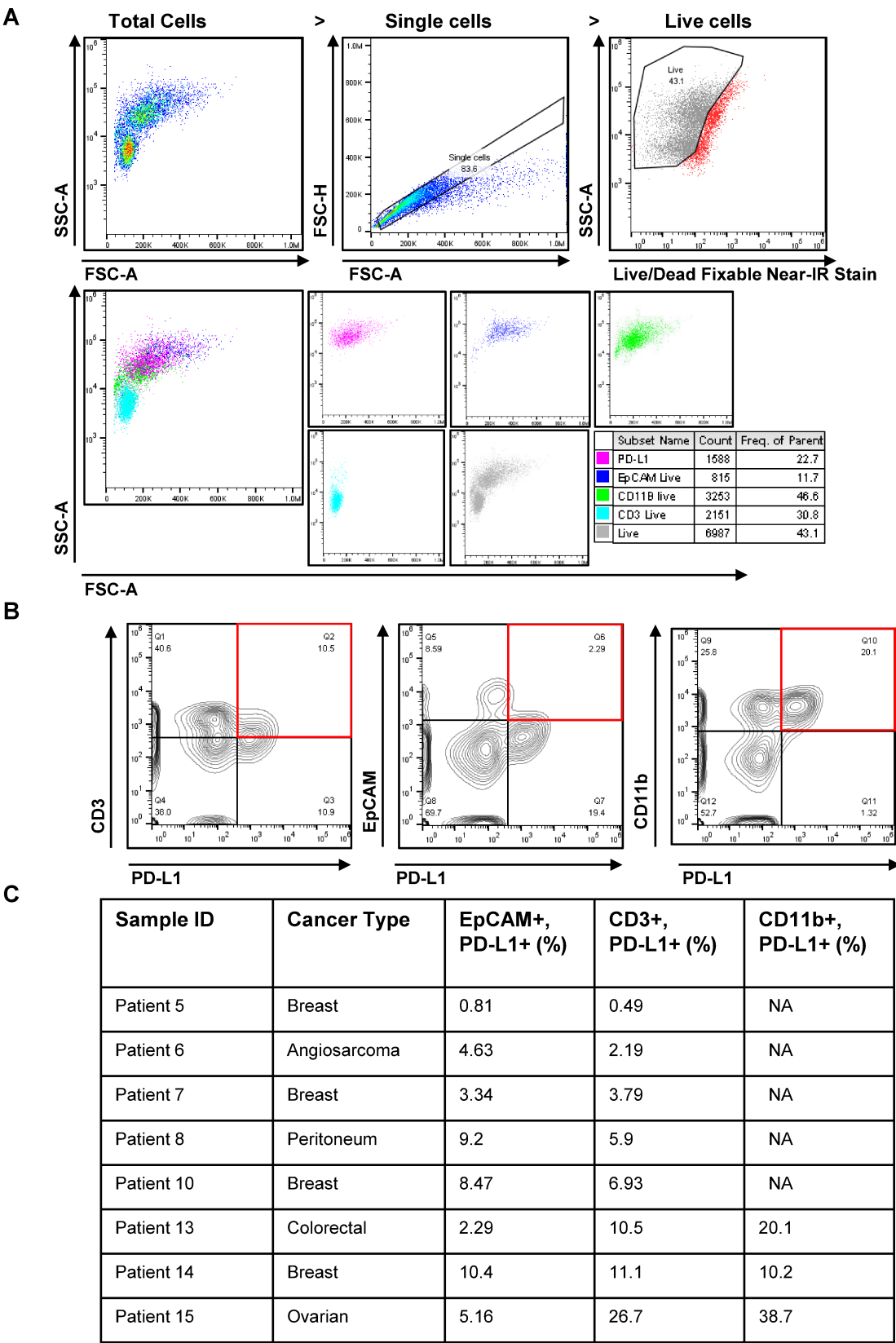

Supplement: Supplementary data [file jitc-2020-001292supp006.pdf]

Supplementary Figure 5

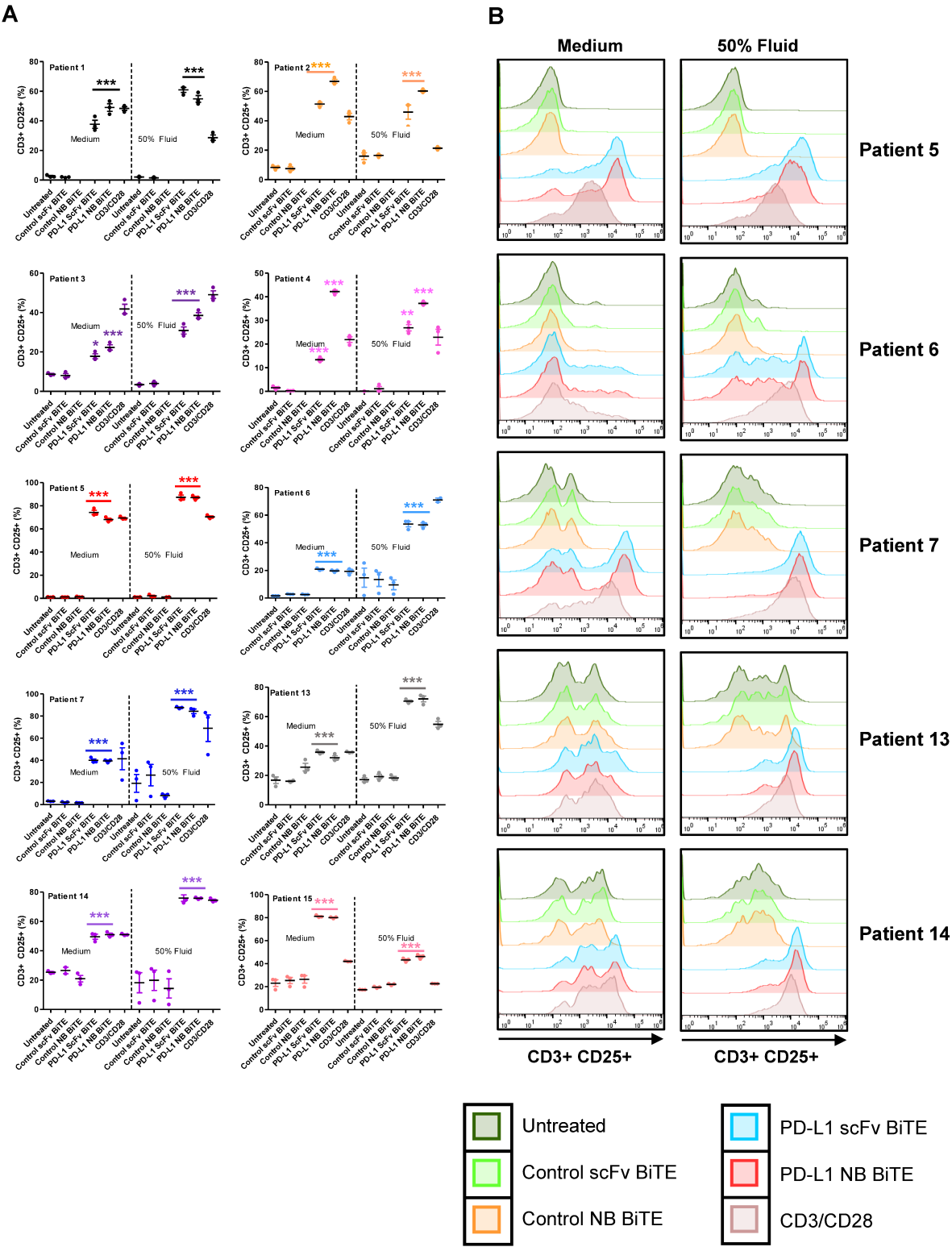

Supplement: Supplementary data [file jitc-2020-001292supp008.pdf]

Supplementary Figure 6

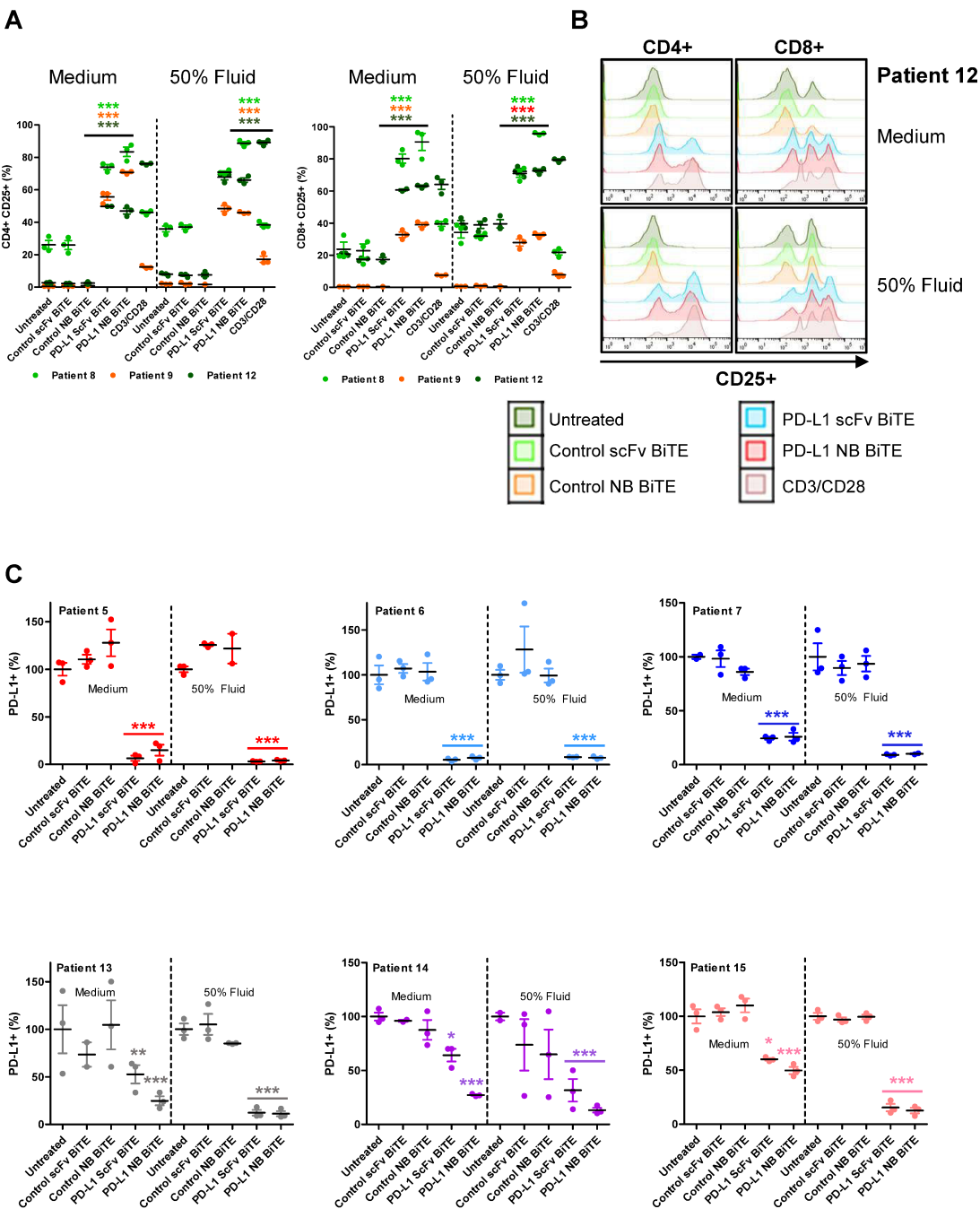

Supplement: Supplementary data [file jitc-2020-001292supp009.pdf]

Supplementary Figure 7

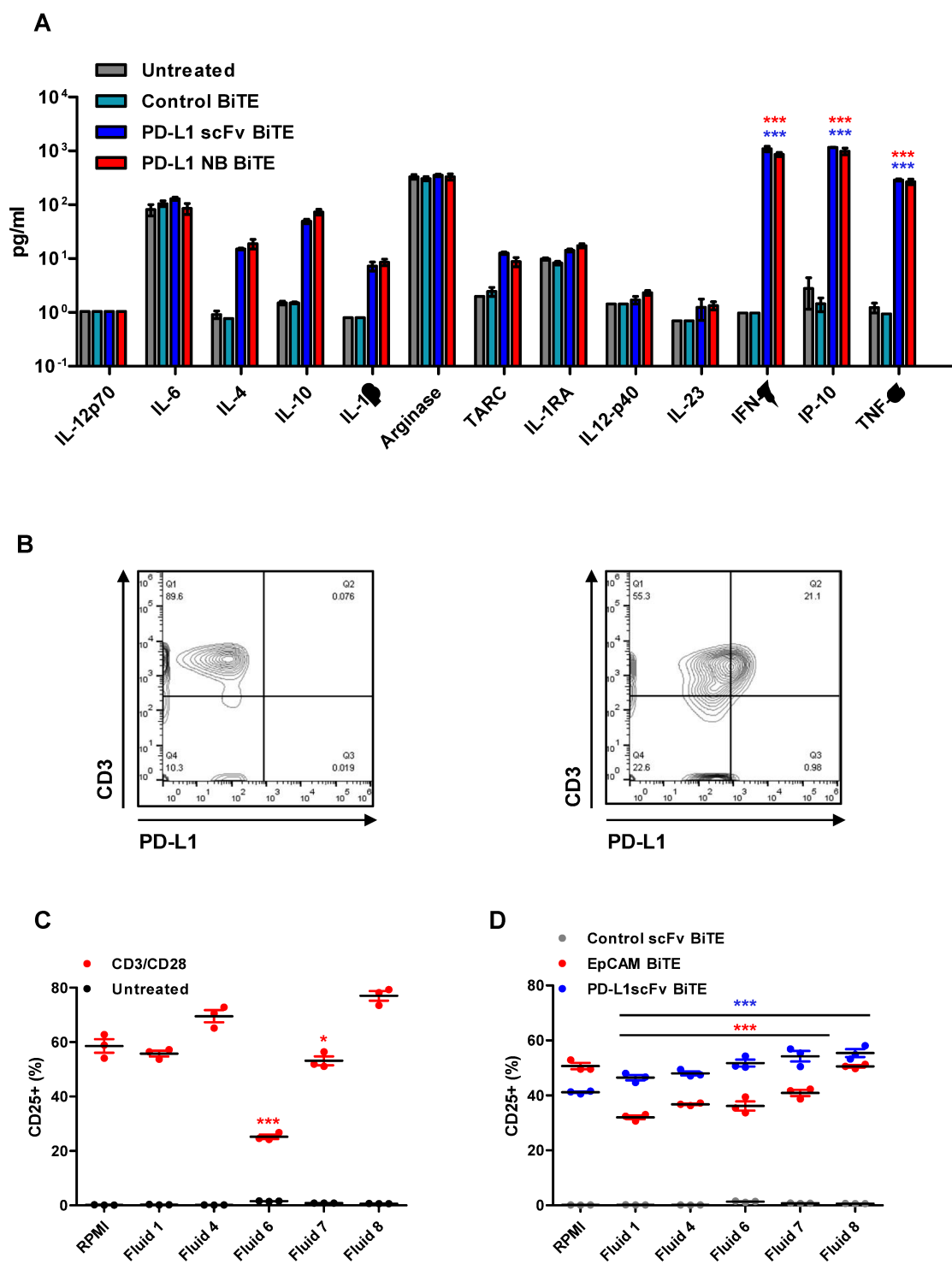

Supplement: Supplementary data [file jitc-2020-001292supp010.pdf]

Supplementary Figure 8

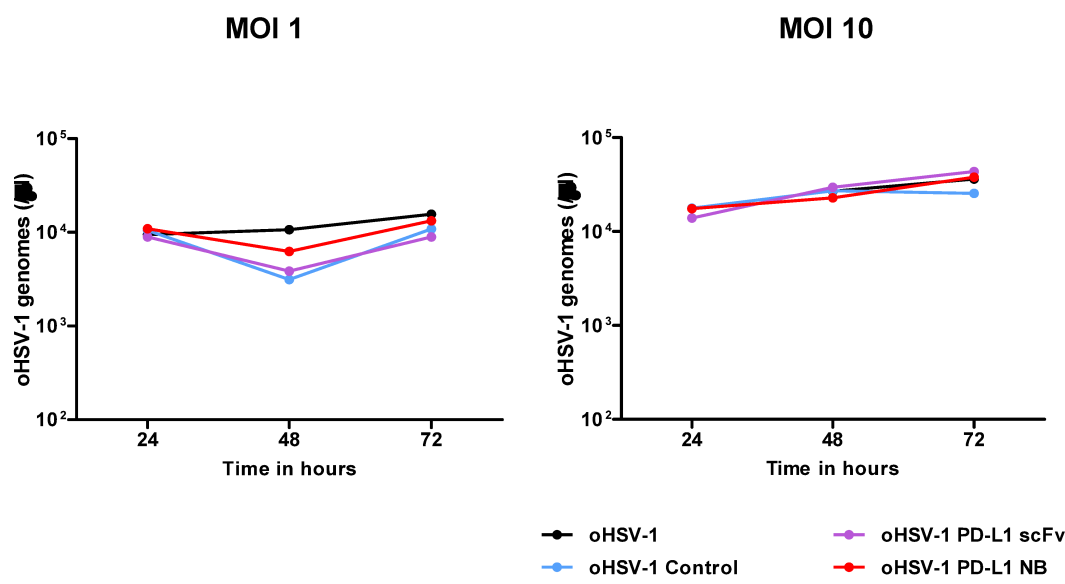

Supplement: Supplementary data [file jitc-2020-001292supp011.pdf]

Supplementary Figure 9

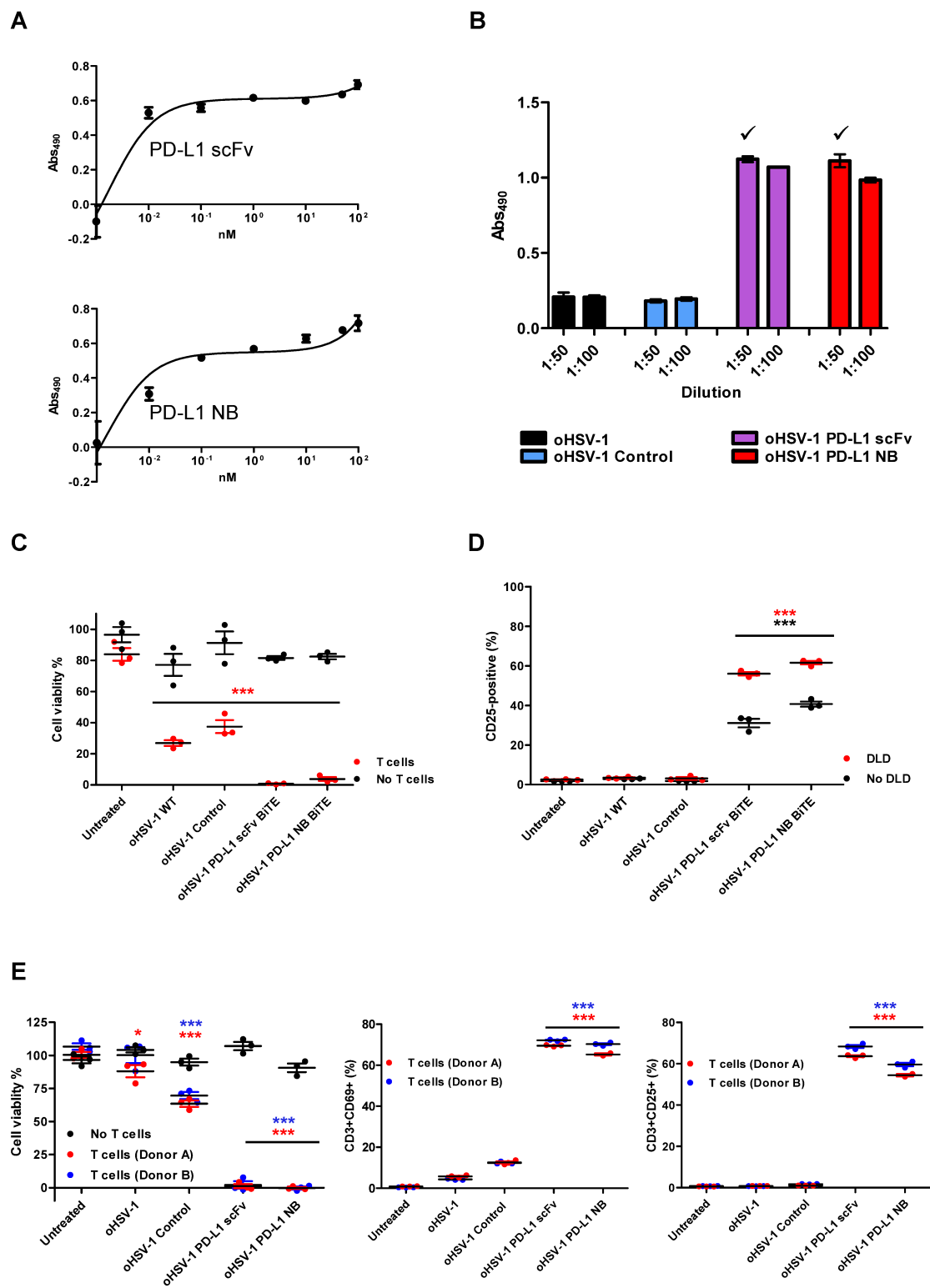

Supplement: Supplementary data [file jitc-2020-001292supp012.pdf]

Supplementary Figure 10

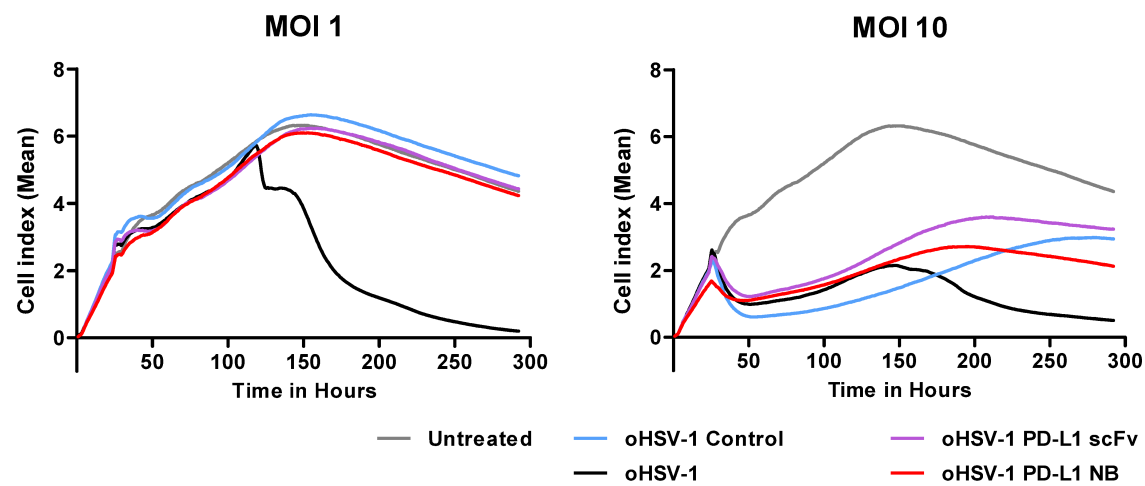

Supplement: Supplementary data [file jitc-2020-001292supp013.pdf]

Supplementary Figure 11

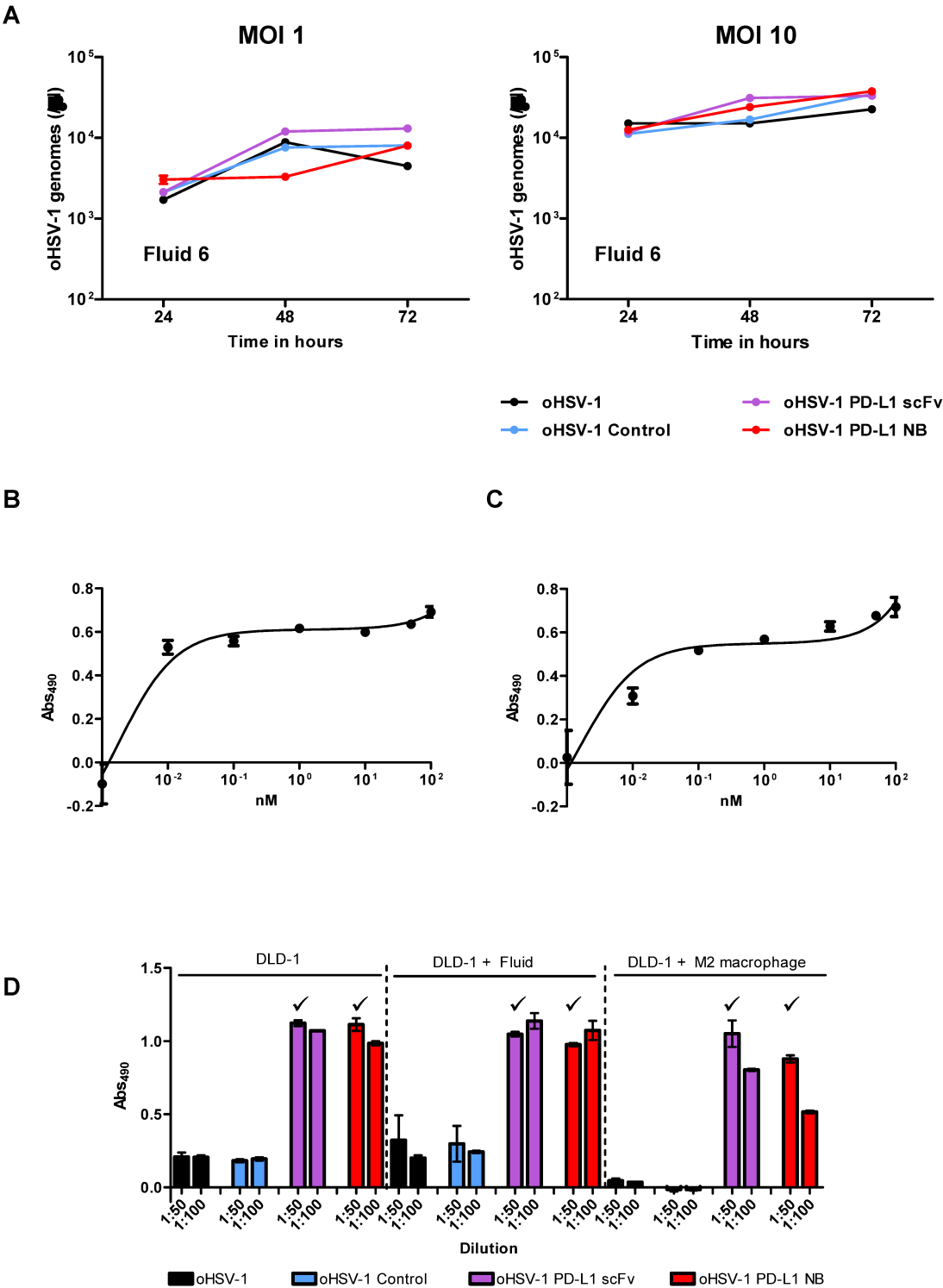

Supplement: Supplementary data [file jitc-2020-001292supp014.pdf]

Supplementary Figure 12

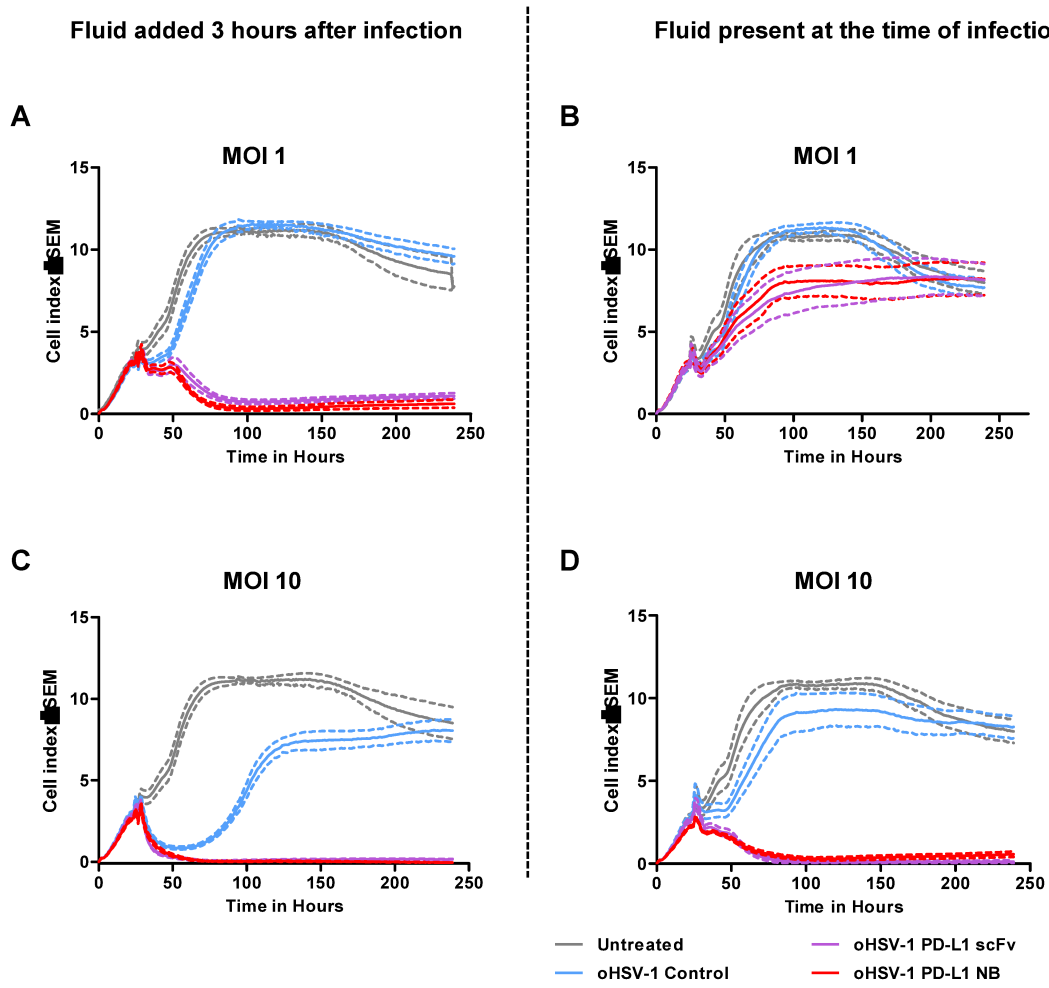

Supplement: Supplementary data [file jitc-2020-001292supp015.pdf]

Supplementary Figure 13

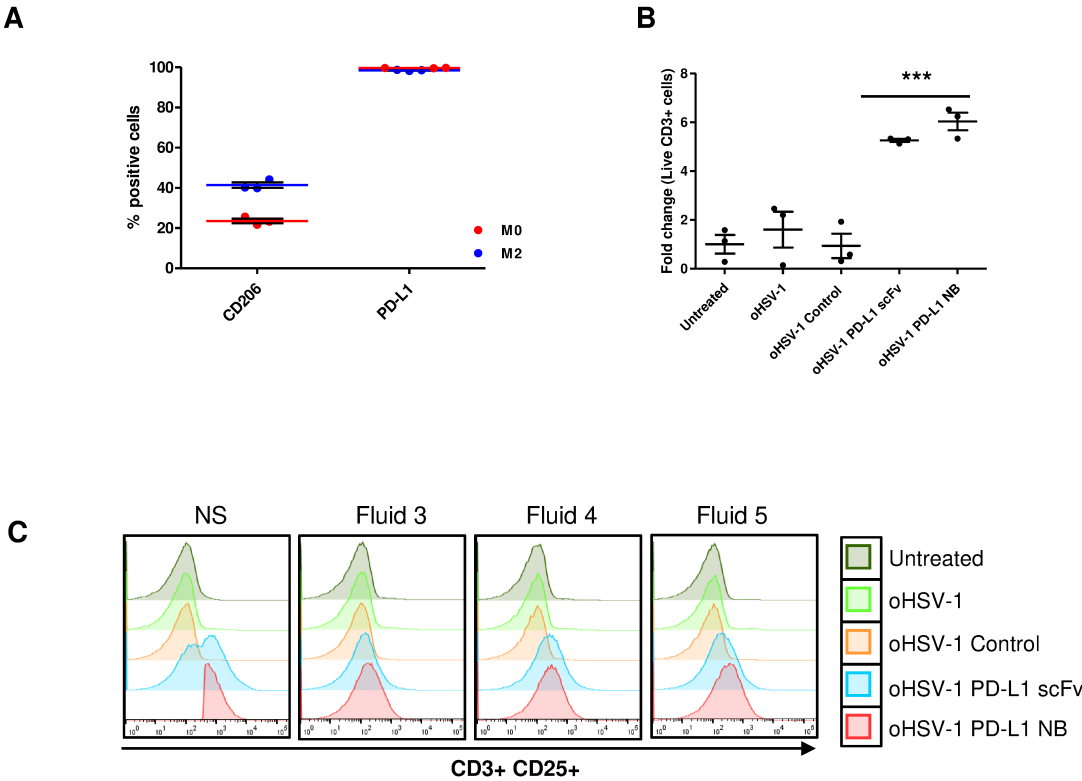

Supplement: Supplementary data [file jitc-2020-001292supp020.pdf]

Supplementary Figure 15

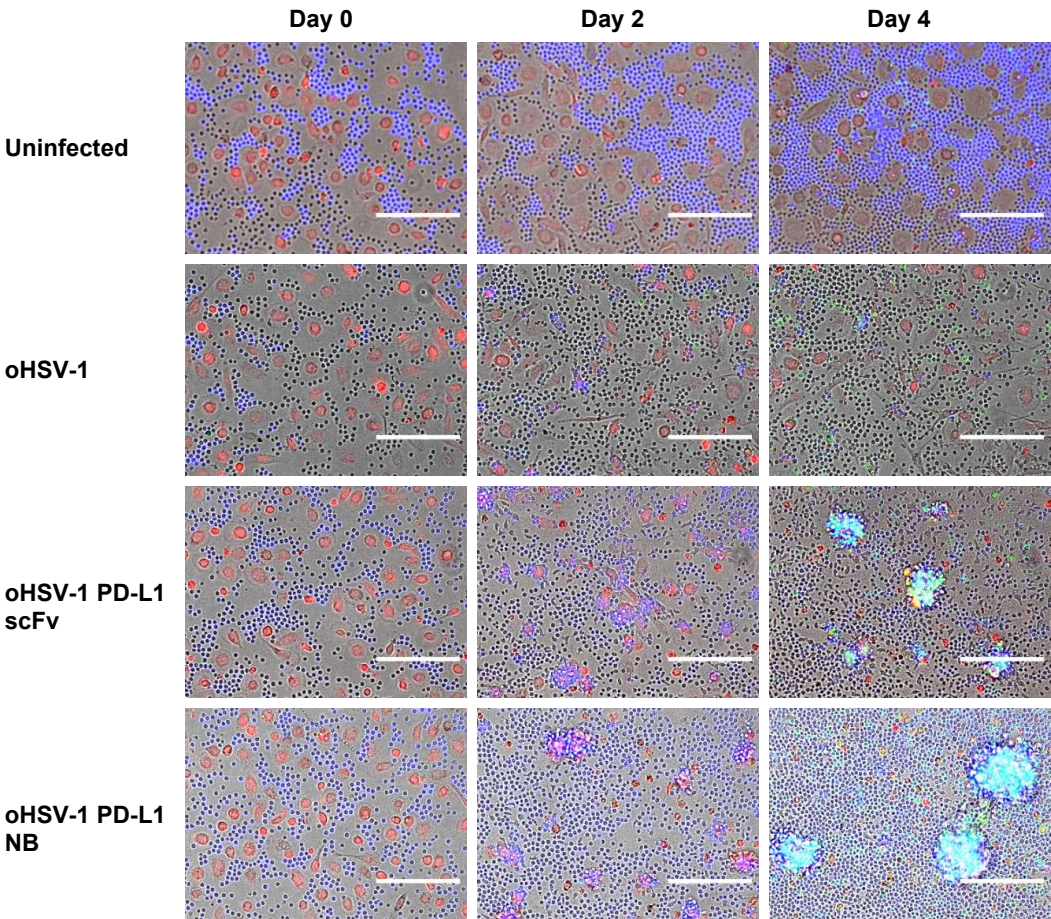

Supplement: Supplementary data [file jitc-2020-001292supp022.pdf]

## Supplementary Figure 16

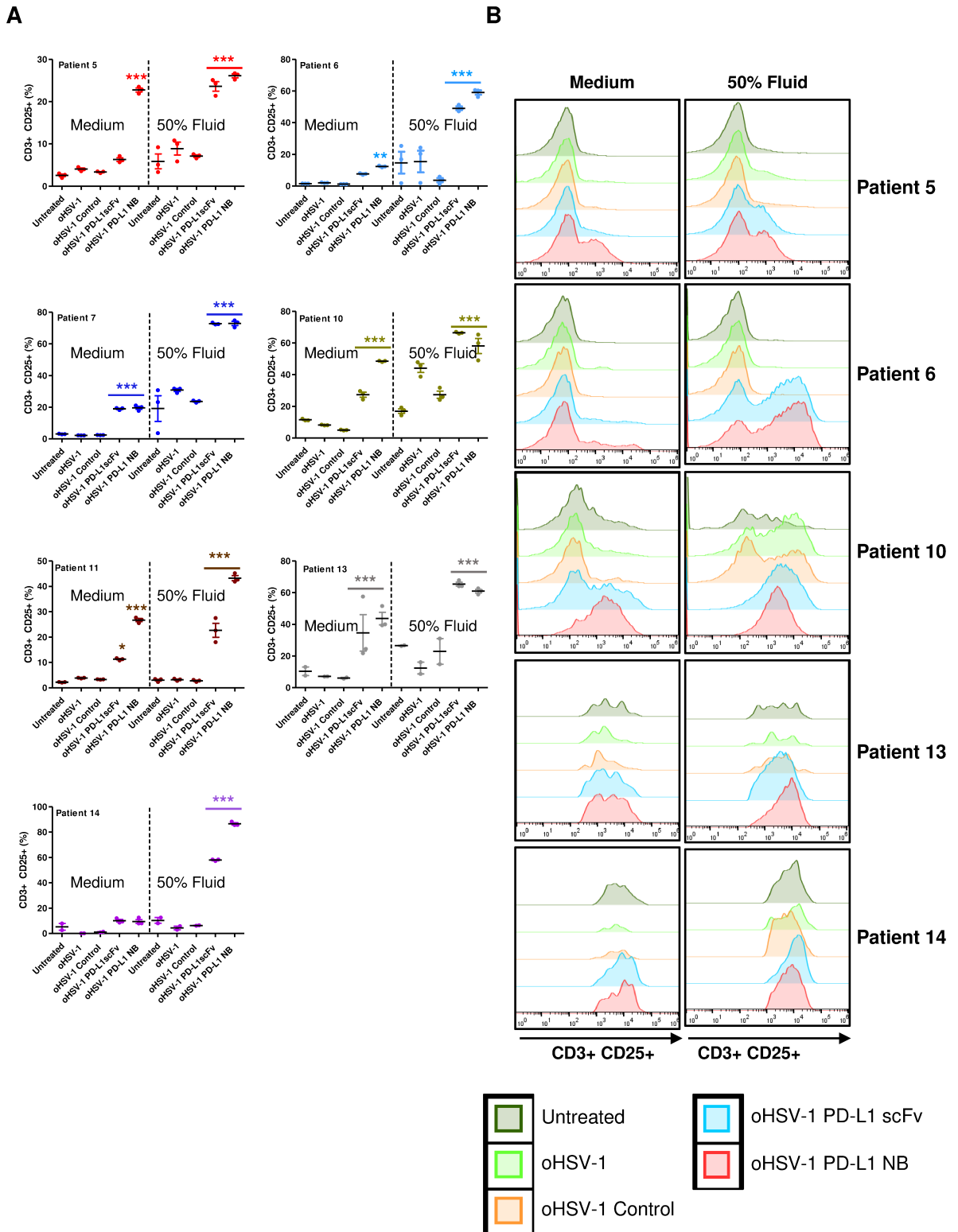

Supplement: Supplementary data [file jitc-2020-001292supp023.pdf]

## Supplementary Figure 17

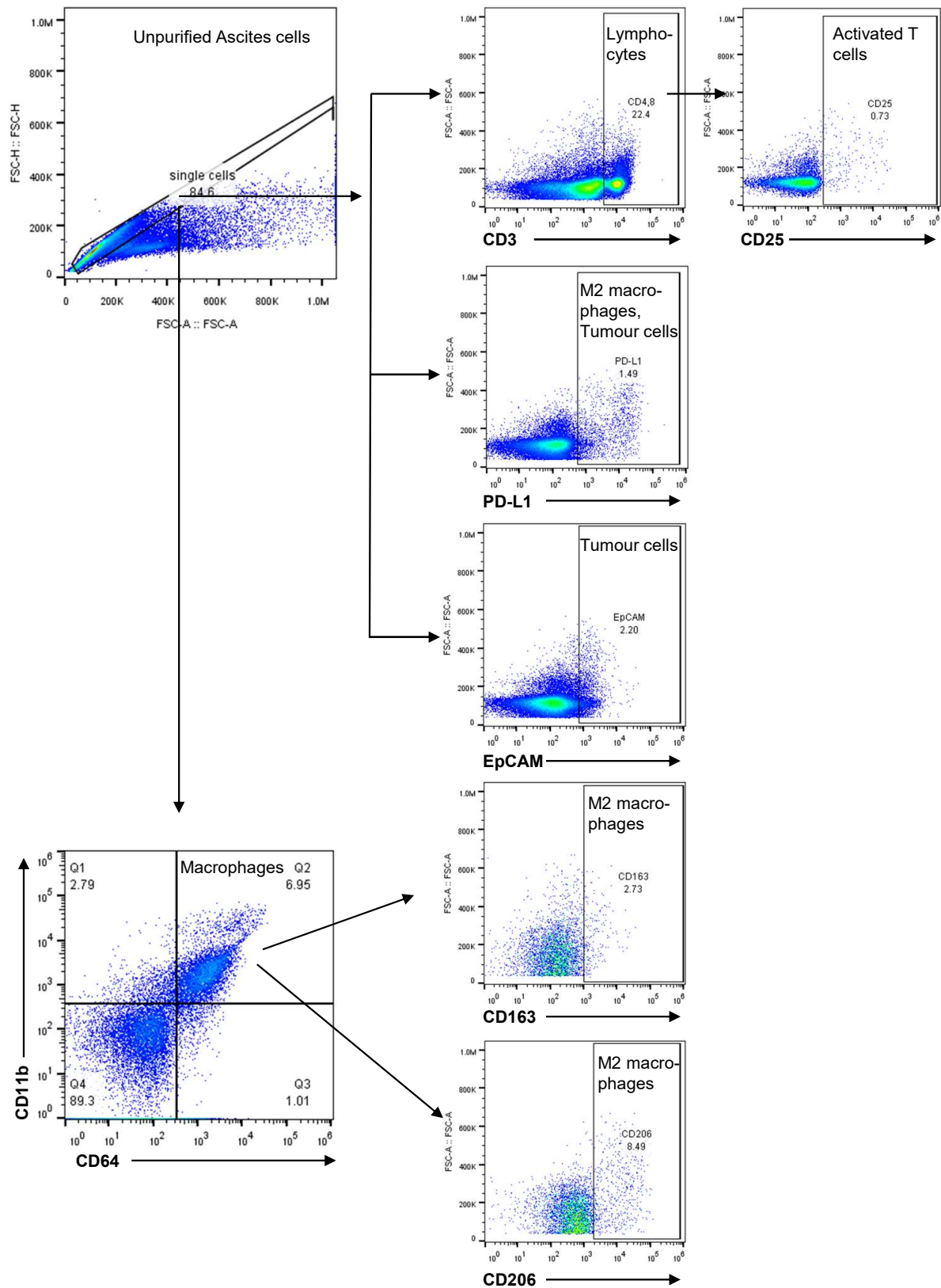

Supplement: Supplementary data [file jitc-2020-001292supp024.pdf]

Supplementary Figure 18

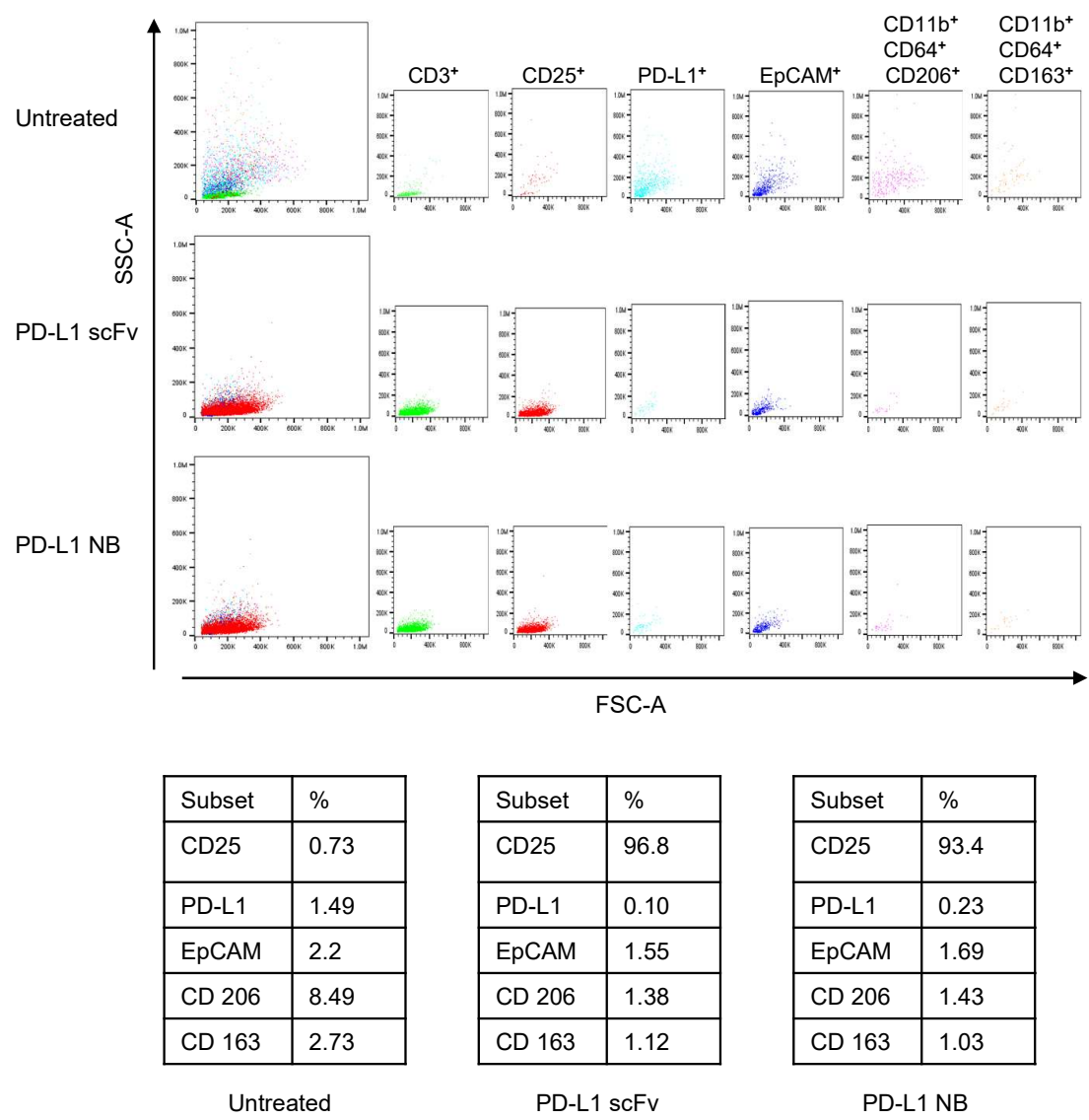

Supplement: Supplementary data [file jitc-2020-001292supp025.pdf]

Supplementary Figure 14

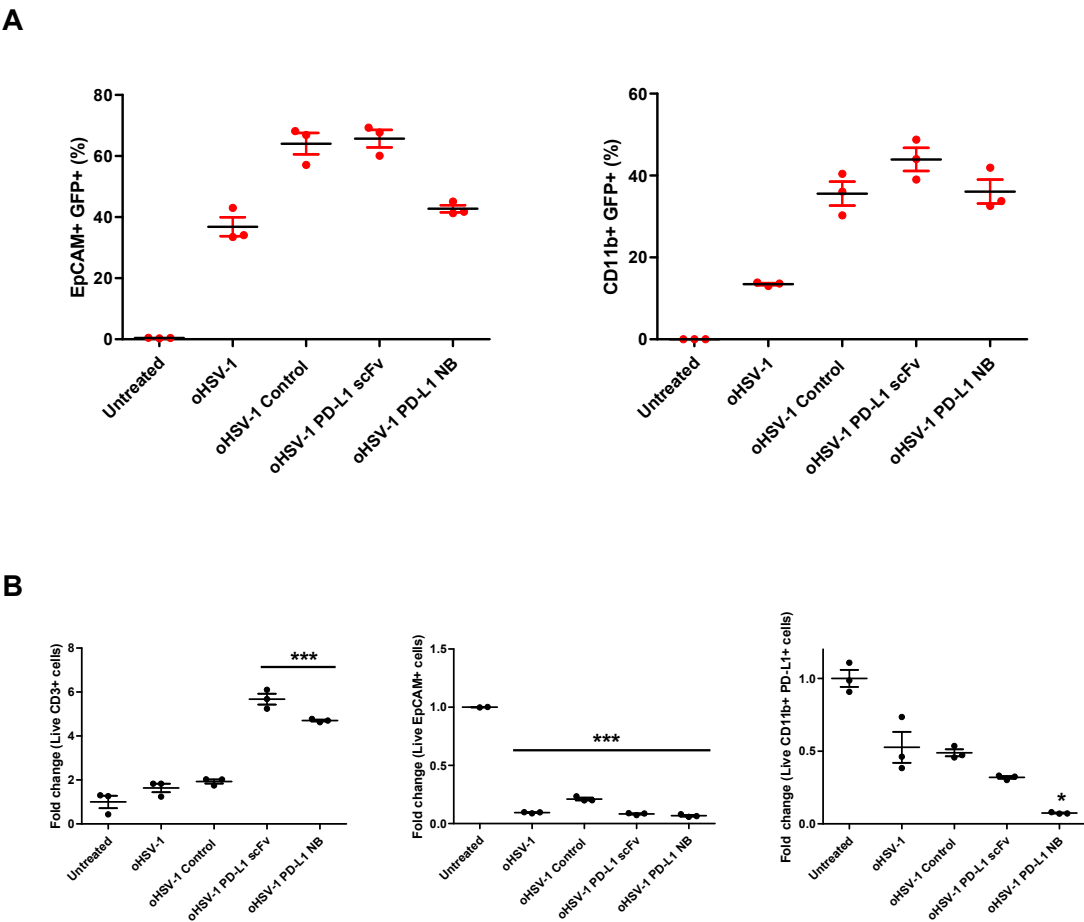

Supplement: Supplementary data [file jitc-2020-001292supp021.pdf]
