# Supplementary material for: Oncolytic herpesvirus expressing PD-L1 BiTE for cancer therapy: exploiting tumor immune suppression as an opportunity for targeted immunotherapy
Source: J Immunother Cancer. 2021 Apr 5;9(4):e001292. doi: 10.1136/jitc-2020-001292 (PMC8026026; doi:10.1136/jitc-2020-001292)
Supplement: Supplementary data [file jitc-2020-001292supp003.pdf]

Supplementary Table-1

Sample	Cancer Type
Patient 1	Ovarian
Patient 2	Gastric adenocarcinoma
Patient 3	Oesophageal
Patient 4	Pancreatic
Patient 5	Breast
Patient 6	Angiosarcoma
Patient 7	Breast
Patient 8	Peritoneum
Patient 9	Breast
Patient 10	Breast
Patient 11	Breast
Patient 12	Ovarian
Patient 13	Colorectal
Patient 14	Breast
Patient 15	Ovarian
Fluid 1	Colorectal
Fluid 2	Ovarian
Fluid 3	Ovarian
Fluid 4	Cholangiocarcinoma
Fluid 5	Pancreatic
Fluid 6	Angiosarcoma
Fluid 7	Breast
Fluid 8	Breast
