# Supplementary material for: Oncolytic herpesvirus expressing PD-L1 BiTE for cancer therapy: exploiting tumor immune suppression as an opportunity for targeted immunotherapy
Source: J Immunother Cancer. 2021 Apr 5;9(4):e001292. doi: 10.1136/jitc-2020-001292 (PMC8026026; doi:10.1136/jitc-2020-001292)
Supplement: Supplementary data [file jitc-2020-001292supp007.pdf]

Supplementary Table-2

Sample	CD3+ (%)	CD4+ (%)	CD8 + (%)	PD-1(% of CD3 or lymphocyte)	EpCAM + (%)	PD-L1+ (%)	CD11b + (%)	CD206 + (%)	CD163 + (%)	FAP+ (%)	CD56+ (%)
**Patient 1**	NA	38.7	NA	19.2	29.3	3.03	32.3	18.8	7.9	NA	29.1
**Patient 2**	NA	35.3	35.6	25.9	5.06	1.28	42.5	47.2	27.5	30.7	NA
**Patient 3**	NA	11.8	15.9	NA	38.6	3.12	NA	14	13.3	NA	2.06
**Patient 4**	NA	44.7	10.2	NA	3.2	0.69	12.6	12.4	3.5	3.41	NA
**Patient 5**	44	8.78	39	2.07 *	3.57	2.41	37.2	42.1	8.66	NA	NA
**Patient 6**	47.1	17.1	22.7	5.46 *	9.18	3.18	38.6	34.5	8.85	NA	NA
**Patient 7**	18.9	11.1	9.59	7.15 *	71.8	1.39	59.9	21.5	3.54	NA	NA
**Patient 8**	NA	14.1	12	NA	48.8	14.8	29.7	21	5.48	10.4	NA
**Patient 9**	NA	35.4	5.95	NA	32.1	29.1	39.2	38.5	23.8	29.6	NA
**Patient 10**	39.80	42.30	8.60	21.30	51.60	6.57	6.48	6.38	3.15	NA	NA
**Patient 11**	40.8	12.2	14.2	3.91 *	5.42	3	43.6	43.6	17.6	NA	NA
**Patient 12**	NA	40.9	27.2	37.3	28.3	0.097	19.7	23.7	4.45	2.58	NA
**Patient 13**	30.8	7.88	15	2.8	11.7	22.7	46.6	NA	NA	NA	7.67
**Patient 14**	7.74	NA	NA	2.58	70.2	16.2	14.8	NA	NA	NA	4.32
**Patient 15**	6.78	14.8	4.61	4.41	28.5	41.4	53.8	NA	NA	NA	5.31

The cell types present in each ascites sample were assessed by flow cytometry and are displayed as a percentage of the total cells in the sample. The percentage of CD3+ cells were estimated by either gating on lymphocyte population (using forward and side scatter; FSC-A and SSC-A) or staining with anti-CD3 antibody. CD4+ and CD8+ population represent percentage of total lymphocytes or CD3+ population. The total percentage of cell exceeds 100% as some cells are positive for more than one marker. NA is shown if the data is unavailable. * represents % of total cells.
